# Natural resistance of tomato plants to Tomato yellow leaf curl virus

**DOI:** 10.3389/fpls.2022.1081549

**Published:** 2022-12-19

**Authors:** Ahmed H. El-Sappah, Shiming Qi, Salma A. Soaud, Qiulan Huang, Alaa M. Saleh, Mohammed A. S. Abourehab, Lingyun Wan, Guo-ting Cheng, Jingyi Liu, Muhammad Ihtisham, Zarqa Noor, Reyazul Rouf Mir, Xin Zhao, Kuan Yan, Manzar Abbas, Jia Li

**Affiliations:** ^1^ Faculty of Agriculture, Forestry and Food Engineering, Yibin University, Yibin, Sichuan, China; ^2^ Genetics Department, Faculty of Agriculture, Zagazig University, Zagazig, Egypt; ^3^ College of Agriculture and Ecological Engineering, Hexi University, Zhangye, China; ^4^ Laboratory Medicine Department, Faculty of Applied Medical Sciences, Umm Al-Qura University, Makkah, Saudi Arabia; ^5^ Department of Pharmaceutics, College of Pharmacy, Umm Al-Qura University, Makkah, Saudi Arabia; ^6^ Key Laboratory of Guangxi for High-quality Formation and Utilization of Dao-di Herbs, Guangxi Botanical Garden of Medicinal Plants, Nanning, China; ^7^ Shaanxi Key Laboratory of Chinese Jujube, College of Life Science, Yan’an University, Yan’an, China; ^8^ College of Horticulture, Northwest A&F University, Yangling, China; ^9^ School of Chemical Engineering Beijing Institute of Technology, Beijing, China; ^10^ Division of Genetics and Plant Breeding, Faculty of Agriculture (FoA), SKUAST–Kashmir, Sopore, India

**Keywords:** Tomato, Tomato yellow leaf curl virus, Ty genes, DNA markers, Molecular responses

## Abstract

Tomato yellow leaf curl virus (TYLCV) is one of the most harmful afflictions in the world that affects tomato growth and production. Six regular antagonistic genes (*Ty-1*, *Ty-2*, *Ty-3*, *Ty-4*, *ty-5*, and *Ty-6*) have been transferred from wild germplasms to commercial cultivars as TYLCV protections. With *Ty-1* serving as an appropriate source of TYLCV resistance, only *Ty-1*, *Ty-2*, and *Ty-3* displayed substantial levels of opposition in a few strains. It has been possible to clone three TYLCV opposition genes (*Ty-1*/*Ty-3*, *Ty-2*, and *ty-5*) that target three antiviral safety mechanisms. However, it significantly impacts obtaining permanent resistance to TYLCV, trying to maintain opposition whenever possible, and spreading opposition globally. Utilizing novel methods, such as using resistance genes and identifying new resistance resources, protects against TYLCV in tomato production. To facilitate the breeders make an informed decision and testing methods for TYLCV blockage, this study highlights the portrayal of typical obstruction genes, common opposition sources, and subatomic indicators. The main goal is to provide a fictitious starting point for the identification and application of resistance genes as well as the maturation of tomato varieties that are TYLCV-resistant.

## 1 Introduction

Tomato (*Solanum lycopersicum* L.) is one of the ubiquitous and vital crops grown worldwide (Howladar, 2016; El-Sappah et al., 2021a; Abbas et al., 2022). The fruit’s appealingness (sizes, colors, flavors, and forms), widespread use, and synthesis of the medicinal chemicals contribute to an annual increase in consumption (Cheng et al., 2020). Tomato fruits are significant because they provide dietary fiber, antioxidants, vitamins, minerals, proteins, carbohydrates, and other nutrients needed for a healthy human diet (Liu et al., 2020; Alluqmani and Alabdallah, 2022). On the other hand, tomato plants are vulnerable to several ailments, and approximately there are 136 viral species known to be harmful (Qi et al., 2021; Islam et al., 2022).

**Figure 1:**
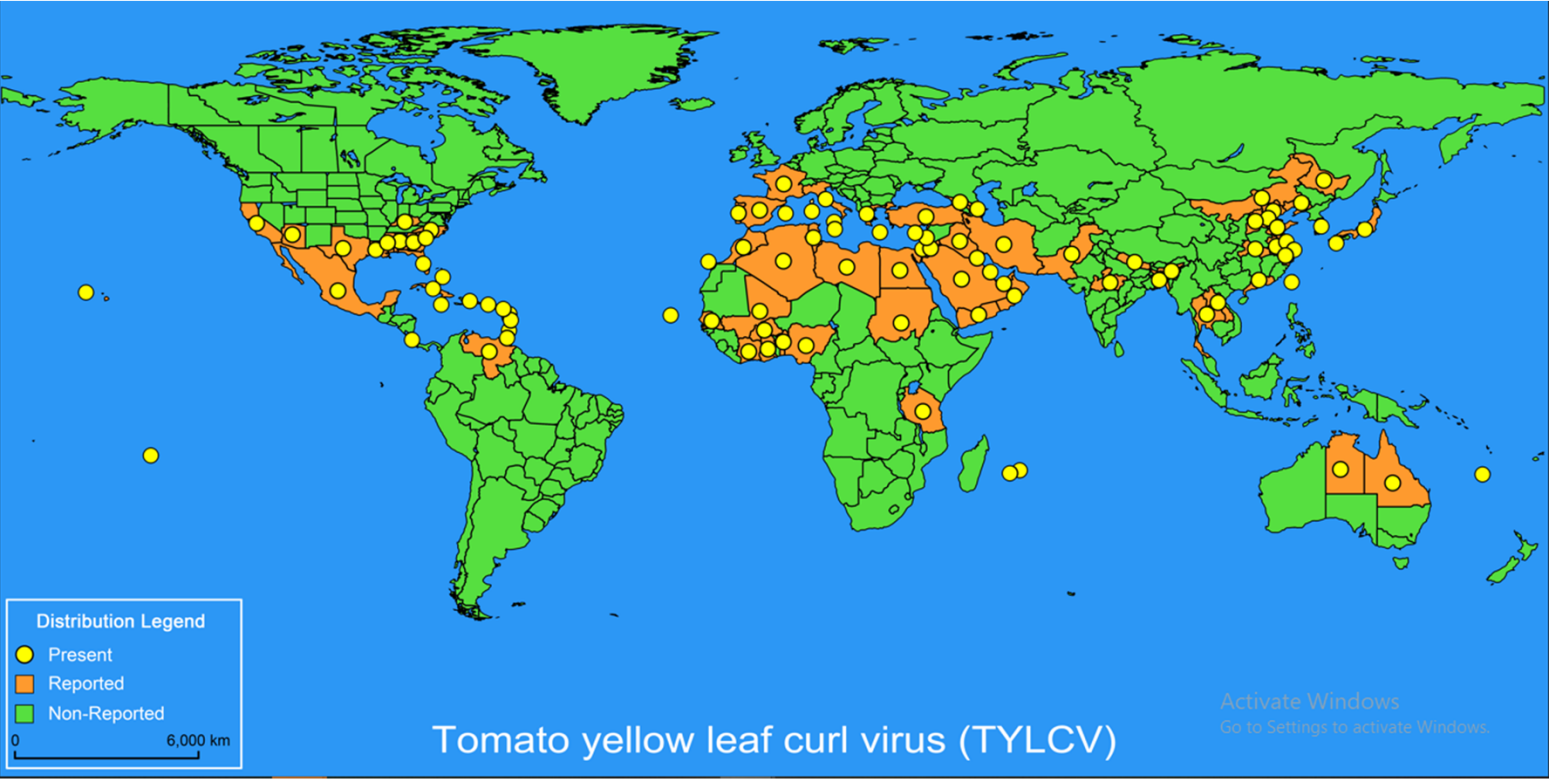
The distribution of Tomato yellow leaf curl virus (TYLCV) globally. The global distribution of TYLCV disease is updated by the EPPO Global Database (19 November 2021). The yellow marks indicate present infection, the orange marks indicate reported condition, and the green marks indicate non-reported infection.

One of the most severe viral diseases to plague tomato plants is tomato yellow leaf curl virus (TYLCV), brought by a group of phylogenetically related *Begomovirus*spp, and spread by the whitefly *Bemisiatabac* (Piedra-Aguilera et al., 2019). TYLCV symptoms in tomato plants are hindered development, chlorosis, leaf curl, and powerless natural product yield (Lapidot and Polston, 2006). In 1932, the TYLCV virus was discovered for the first time in Sudan and the Middle East (Idris and Brown, 2005; Van Brunschot et al., 2010). It has since spread throughout the world’s tropical and subtropical regions, including the Mediterranean Basin, the Far East (Asia), the Caribbean, Australia, North, South, and Central America, and many others (**Figure 1**) (Czosnek and Laterrot, 1997; Navas-Castillo et al., 1997; Zhang et al., 2009; Péréfarres et al., 2010; Ning et al., 2015; Kil et al., 2016). Recently, it was discovered that TYLCV was seed-distributed (Kil et al., 2016; Kil et al., 2017; Kil et al., 2018). However, the vector *Bemisiatabaci* (Gennadius) (Hemiptera: Aleyrodidae), notably biotypes B and Q, are responsible for the majority of diseases during transportation (Pan et al., 2012) by the whitefly in its adult stage, *Bemisiatabaci*, possibly the most bothersome pest of vegetables and other produce (Ning et al., 2015; Pakkianathan et al., 2015).

Diverse methodologies have been utilized to hinder the spread of TYLCV, including strict quarantine guidelines, traditional rearing, and hereditary designing (Lapidot and Polston, 2006; Polston and Lapidot, 2007). Till today, cultivated tomato varieties have included the *Ty-1* to *Ty-6* resistance genes from their related species, which has resulted in the development of virus resistance, but it’s crucial to emphasize that this resistance has never been entirely effective (Prasad et al., 2020; Yan et al., 2021; Mori et al., 2022). *Ty-1*, *Ty-2*, *Ty-3*, and *ty-5* have all been cloned in recent years (Ren et al., 2022). *Ty-1, Ty-3, Ty-4*, and *Ty-6* genes were all introduced from *S. chilense*accessions and are located on chromosomes 6, 6, 3, and 10, respectively (Zamir et al., 1994; Ji et al., 2007a; Ji et al., 2009b; Kadirvel et al., 2013; Tabein et al., 2017; Gill et al., 2019). The *ty-5* and *Ty-2* were introgressed from *S. peruvianum* and *S. habrochaites*, respectively (Hanson et al., 2006; Hutton et al., 2012). *Ty-1* is an allelic variant of *Ty-3* that codes for an RNA-dependent RNA polymerase; it is the first resistance locus discovered in *S. chilense *(LA1969) (Verlaan et al., 2013). The *Ty-1* can make geminiviruses more resistant by boosting the viral genome’s cytosine methylation (Butterbach et al., 2014). The *Ty-2*, a dominant resistant gene on chromosome 11, can confer resistance to some monopartite begomoviruses, including TYLCV, but not to any other monopartite or bipartite begomoviruses like Tomato yellow leaf curl Sardinia virus (TYLCSV) (Barbieri et al., 2010; Prasanna et al., 2015). *Ty-4* confers resistance to TYLCV less effectively than *Ty-3*, which was delivered by introgression from the long arm of chromosome 3 of *S. chilense* (LA1969) (Ji et al., 2009b). The tomato homolog of the messenger RNA surveillance factor *Pelo*, implicated in the ribosome recycling phase of protein synthesis, has recently been linked to the *ty-5* gene (Anbinder et al., 2009), located on chromosome 4 of *S. peruvianum* (Lapidot et al., 2015). The *Ty-6* gene confers resistance against monopartite and bipartite begomoviruses, completing the protection provided by the known *Ty-3* and *ty-5* genes (Gill et al., 2019).

The resistance breakthrough fueled the TYLCV dispersion, prompting plant breeders to continually search the wild tomato gene pool for potent new sources of resistance. Immunization schedules, screening and validation of resistance sources, gene discovery and genetic mapping, field evaluation of resistance gene transfer to cultivars and inbred lines, global dissemination, TYLCV symptoms, and immunization techniques are all part of TYLCV resistance breeding programs. The presentation will focus on innate immunity to TYLCV, organized resistance genetic defense, marker-assisted resistance breeding selectable markers, and natural resistance resources. The main objective is to lay the background for future studies into the genes responsible for TYLCV disease resistance.

## 2 TYLCV symptoms in tomato plants

**Figure 2:**
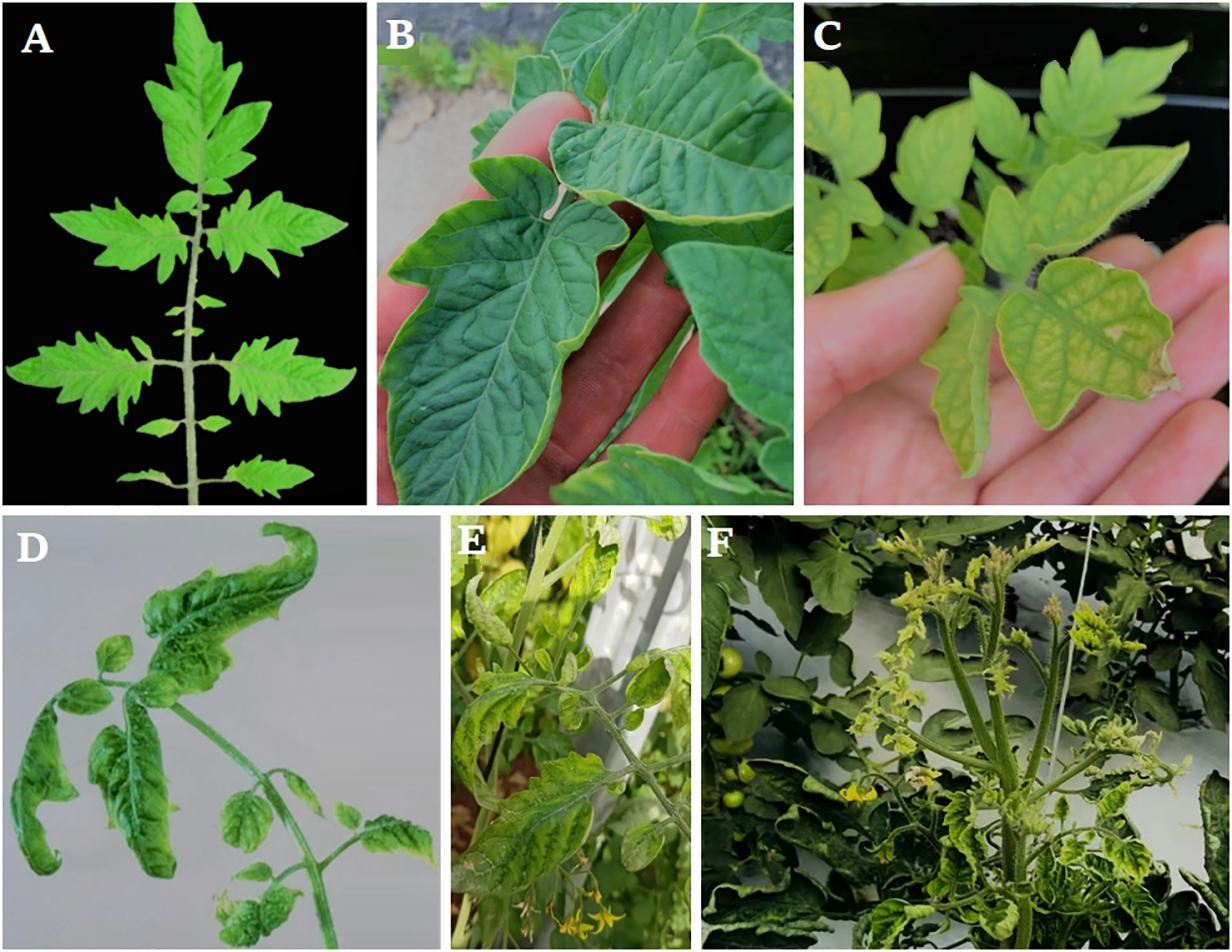
Typical symptoms of TYLCV, **(A)** Normal plant showed no infection, **(B)** Infection at a very early stage between 2 to 4 weeks post-infection, **(C)** tomato plant after five weeks infection, **(D–F)** Infection at a late stage where the plants show severe symptoms consisting of marked yellowing, puckering, and severe size reduction in the top leaves.

Earlier study shows that the whole tomato plants were weakened by TYLCV infection, and there was a significant decrease in the yield. After inoculation, the first TYLCV symptoms on tomato plants appear 2-4 weeks later and may take up to two months to fully manifest (Ioannou, 1985). The virus isolates, host genetic background, ambient conditions, development stage, and physiological state of the tomato plant at the time of infection influence the kind and intensity of symptoms (Mauck, 2016). The leaflets of newly emerged leaves curl downward and inward in a hook-like pattern early after infection (Salati, 2001) (**Figure 2**). Afterward, leaves are distorted and narrower with chlorosis in the center and edges and leaflet margins curling upward (Vallad et al., 2018). The underside of leaflets may also be stained purple (Gorovits et al., 2013; Ibne-Siam-Joy, 2020).

Temperatures exceeding 25°C can aggravate leaf symptoms (Polston and Lapidot, 2007; Anfoka et al., 2016). Infectious plants showed severe symptoms such as yellowing, curling, and a significant loss in apical leaf size (Lapidot et al., 2001). The leaves curl between the veins, and the midrib may become arched And thepetioles twisting can be seen in elder leaves (Shankar et al., 2014). The leaf surface was found to have pale yellow dots that grew larger with time (Gorovits et al., 2013) which is, however, a less prevalent symptom. Tomato plants contaminated with TYLCV were significantly stunted, with numerous branchlets and reduced internodes (Davino et al., 2006; Shankar et al., 2014). Due to increased flower shedding, young, early-affected plants are frequently infertile. Since most blooms (>90%) droop after infection, there is almost no fruit. As a result, yield reductions are more significant when plants are infected prematurely. Late-stage infections can drastically diminish the yield of new fruit. Infected plants produce fewer and smaller fruits that come off easily. Fruit ripens appropriately and is likely to bear before infection (Dam et al., 2005).

## 3 Methods for identifying resistance to TYLCV in tomato

**Figure 3:**
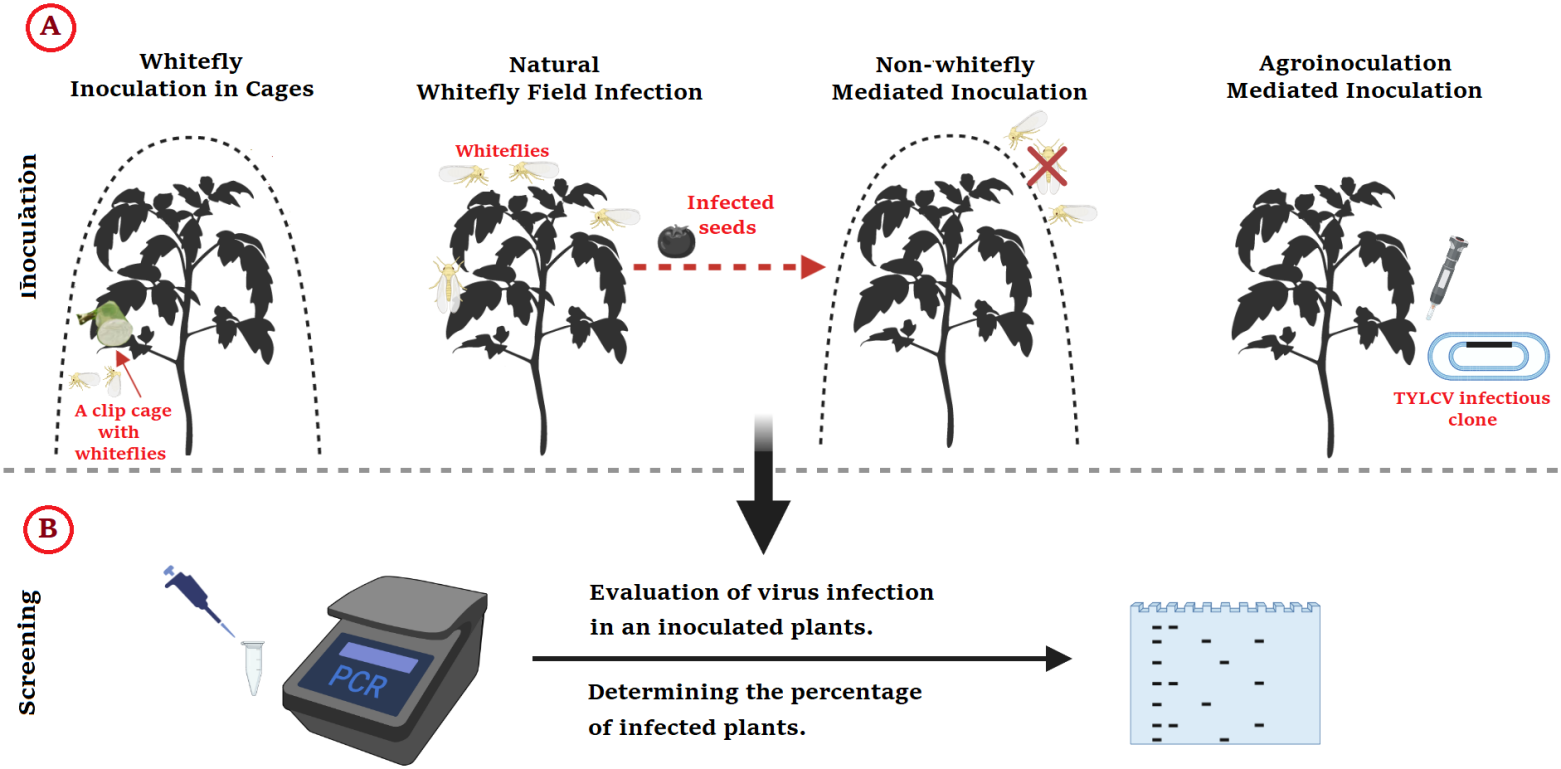
Methods for Identifying Resistance to TYLCV in Tomato; **(A)** Inoculation types, followed by **(B)** PCR screening method. This figure was made using BioRender.

*In vivo* and *in vitro* approaches are used to test the rate of TYLCV infection and understand the processes of plant resistance to TYLCV as shown in **Figure 3**.

### 3.1 *In vivo* infection

#### 3.1.1 Natural whitefly field infection

It is a method for the testing of TYLCV resistance and essential to recognize hotspots and favorable seasons for effective screening under natural disease conditions (Dhaliwal et al., 2020). The two types of field infection are controlled greenhouse inoculations and spontaneous field inoculations (Lapidot, 2007). Even under intense inoculation pressure, many plants resist infection, proving that spontaneous field infection is frequently ineffectual (Vidavsky et al., 1998). Only 50% of susceptible tomato plants with whiteflies and TYLCV infections became infected within the first month of germination in whitefly and TYLCV-infested areas. Only 10% of sensitive plants survived the infection 90 days after transplantation despite rising whitefly numbers and easily accessible viral inoculums (Vidavsky et al., 1998). Another study by Cohen et al. (1988) discovered that the percentage of virus-carrying whiteflies in the general whitefly populace in the field was deficient. Only 3-6 percent of whiteflies obtained in the wild can spread the virus based on the TYLCV-sensitive host from which they were taken.

It should be emphasized that a sensitive plant evading inoculations may be employed as a resistant parent in subsequent crosses if it is (inadvertently) checked for resistance. A deluge of supposedly resistant yet sensitive plants can quickly jam breeding operations. Therefore, choosing tomato plants only based on an infected field’s lack of symptoms may be deceiving (Vidavsky et al., 1998). In addition to facilitating inoculate outbreaks, spontaneous field inoculations have other drawbacks. Due to late and asynchronous infection, inoculations may result in less severe disease symptoms than controlled inoculations. (Picó et al., 1998). Compared to plants infected at a younger age, plants inoculation at a mature age may have milder symptoms. Milder symptoms could be misconstrued as signs of genetic resistance instead of just late infection. Several TYLCV-resistant cultivars respond sensitively to controlled greenhouse inoculations, whereas field inoculations result in resistance levels comparable to other, more resistant cultivars (Picó et al., 1998). The response of resistance sources to TYLCV may differ depending on the inoculation technique used, with controlled greenhouse inoculations corresponding to high inoculum concentrations and spontaneous field inoculations corresponding to low inoculum concentrations. The most sensitive genotypes might be excluded by field testing, despite the low and delayed incidence of illness following spontaneous field inoculations (Picó et al., 1998; Picó et al., 2000; Lapidot, 2007). Another issue with spontaneous field inoculations is that TYLCV-resistant plants could incorrectly be labeled sensitive after contracting unrelated viruses or other diseases. Whitefly stress, inoculation intensity, viral inoculum size, and plant age were unknown variables in field the inoculations. Also unknown is the interval between getting the whitefly and the virus’s propagation. Like all begomoviruses, TYLCV is spread *via* a sustained, cyclical process from its whitefly vector. Although transmission can continue for the duration of the vector’s existence, transmission efficiency degrades over time, as demonstrated in TYLCV (Cohen and Harpaz, 1964; Picó et al., 1998; Lapidot et al., 2001).

Consequently, it is unknown and impossible to replicate the effectiveness of field inoculations. Several metrics can be used to compare inoculation effectiveness in controlled greenhouse inoculations to spontaneous field inoculations. The researchers administering the inoculations during controlled greenhouse can change the whitefly age, feeding time, transfer nutrition time, number of whiteflies per infected plant, and so on. It should be remembered that when used as viral vectors, whiteflies can cause significant damage to plants as well as transmit viruses. Whiteflies devour plants by over-juicing, secreting honeydew (which encourages the formation of black mold), and creating systemic illness (Schuster et al., 1990; Byrne and Bellows, 1991). Whitefly inoculations feed on target plants, therefore, need long enough to include successful inoculations but short enough to limit direct whitefly harm. After exposing young tomato seedlings to a large number of virus-carrying whiteflies (about 30-50 whiteflies per plant, which transmit the virus to plants by feeding with almost 100% efficiency) for 48 hours (commercially acquired feeding), all sensitive controls were infected with TYLCV (Lapidot et al., 1997).

#### 3.1.2 Whitefly inoculation in cages

For TYLCV screening, whitefly-mediated mass inoculation or individual plant inoculation is commonly used. Non-preference issues could arise even with carefully regulated greenhouse inoculations. Whiteflies prefer to feed on other tomato plants when inoculating various plants in the same spot (Legarrea et al., 2020). They are inefficient at inoculating one specific type or variety and dislike most physical obstacles in tomatoes, including waxy or thick cuticles or specific trichomes, which hinder whiteflies from colonizing and feeding on those leaves (Bellotti and Arias, 2001). When inoculating wild tomato cultivars, this issue is most apparent. Adding some wild species seems to help prevent infection when looking for new sources of resistance because they are not preferred by whiteflies (Picó et al., 1998). One inoculation of many wild tomato cultivars in cages can solve the non-preference issue. In this instance, a plant is put in a cage with virus-carrying whiteflies, forcing them to feed on the target plant because it is the only plant they will consume (thus spreading the virus). Precision work, or the lack thereof, is another issue that has emerged since the widespread administration of the whitefly vaccine. It is practically impossible to determine the proper number of whiteflies per plant and which leaves to target for inoculation when vast numbers of plants are infected on a big scale. Whiteflies can be precisely controlled using clip cages or leaf cages. Managing the number of whiteflies used per plant, their age and gender, the precise length of the acquisition access period (AAP) and the inoculation access period (IAP), and the TYLCV inoculation location are all advantages of clip cage inoculation (Lapidot, 2007). Because cages allow all test plants to employ the same whitefly-mediated inoculation circumstances, they also make it possible to compare how various plants react to TYLCV infection, for instance, when comparing plants with varying degrees of viral resistance. The clip cage is a tiny cylinder of clear plastic with both sides clipped off. One side of the cage has a thin mesh cover that may be opened to suck whiteflies into the cage. A clip may quickly fasten to the opposite side’s bottom of the preferred wing. The AAP claims that physical barriers like gills, waxy or thick cuticles, or the presence of certain trichomes that prohibit whiteflies from colonizing and feeding on such leaves are to blame for a known number of whitefly-by-whitefly epidemics (Bellotti and Arias, 2001).

#### 3.1.3 Non-whitefly-mediated inoculation

Other non-whitefly-mediated inoculation techniques are required because whiteflies must be bred to improve a time-consuming and laborious TYLCV-controlled inoculation protocol (Kil et al., 2016; Dhaliwal et al., 2020). Mechanical TYLCV transfer has been attempted using various sources and test plants (Lapidot, 2007). When using datura (*Datura stramonium*) plants as parent plants, the highest mechanical TYLCV transmission rates only reached less than 17%. Only 12% of tested plants, such as the datura, were successful (Dhaliwal et al., 2020). The transmission was not achieved during mechanical inoculation utilizing tomato plants as the parent and test plants (Makkouk et al., 1979). As a result, even though TYLCV can be mechanically transmitted, the success rate is too low to support the creation of a successful inoculation regimen using this technique. To immunize against TYLCV, inlay inoculations are used. In this technique, the test plants are either grafted onto scions infected with TYLCV or laterally with the infected plants’ leaves or tips (Pereira-Carvalho et al., 2015; Koeda et al., 2020). Graft inoculation has been used to identify plants resistant to TYLCV with increasing transportation effectiveness (Pico et al., 2001; Leibman et al., 2015). The characteristic of graft inoculation is the ongoing exposure of test plants to the highest concentrations of viral inoculums. Grafting is a method that can be used to test resistance. When resistant plants were grafted with TYLCV-symptomatic leaves, the plants remained symptom-free (Friedmann et al., 1998). (Kegler, 1994; Friedmann et al., 1998). Graft inoculation is ineffective as a mass inoculation technique because it is labor-intensive and time-consuming (Sastry and Zitter, 2014).

### 3.2 *In vitro* infection/agroinoculation

Agroinoculation inoculation is yet another TYLCV inoculation test (Czosnek et al., 1993; Kheyr-Pour et al., 1994; Lucioli et al., 2003; Mori et al., 2021). *A. tumefaciens* is employed for agroinoculation to introduce cloned viral DNA into host cells (Grimsley et al., 1986; Lu et al., 2003; Delbianco et al., 2013). Tandem repeats (or 1.5–1.8 -mers) of the viral genome are cloned into the T-DNA of the Ti plasmid of *A. tumefaciens* in the case of TYLCV and another geminivirus before being injected into plants. Replicates are widespread systemically in plants due to genome-sized viral DNA forms, which also cause disease symptoms (Stenger et al., 1991). Agroinoculation is a popular technique used to inoculate plants or leaf discs with geminiviruses. It has been suggested that agroinoculation be utilized as a test technique for TYLCV inoculation and screening of resistant plants because it has been used successfully to introduce the virus into leaf discs and entire plants (Czosnek et al., 1993; Kheyr-Pour et al., 1994). However, it has been demonstrated that cloned TYLCV DNA delivered through agroinoculation inoculation can surpass the virus’s natural resistance in wild tomato species (Abhary et al., 2006; Maliano, 2021).

The effectiveness of agricultural immunization for testing of TYLCV resistance was shown to be dubious (Kheyr-Pour et al., 1994). The usefulness of agricultural immunization as a technique to test various wild and farmed tomato genotypes for TYLCV resistance was recently examined (Pico et al., 2001). Rub agroinoculation (rubbing emery-dusted leaves with an *Agrobacterium tumefaciens* suspension) led to irregular, weak infections and failed to distinguish between genotypes with various levels of resistance (Pico et al., 2001; Lapidot, 2007). Although the inoculation rate of susceptible controls was 100%, the inoculation efficiency of resistant genotypes was lower. Agrobacterium inoculation of the strain (injection of *A. tumefaciens* suspension into the strain) was more successful (Lapidot, 2007). It was determined that agricultural immunization might be employed in breeding programs but only as an adjunct to immunization against whiteflies (Pico et al., 2001).

Plants have also been vaccinated with Bergomo virus DNA using particle bombardment (gene gun inoculation) (Garzón-Tiznado et al., 1993). Biolistic Inoculation of cloned Bergomo virus DNA per unit-length (monomer) or tandem repeats (dimer) results in high inoculation efficiency by removing time-consuming DNA manipulation and enabling the inheritance of bergomovirus analysis (Bonilla-Ramírez et al., 1997). Although biolistic inoculation of viral DNA monomers still presents in the cloned plasmid has been proven to be possible, it has only been accomplished after the viral clones removed from the plasmid increased vaccination rates (Liu, 2011; Ceniceros-Ojeda et al., 2016).

However, biolistic inoculation has only been observed in begomoviruses: only the tomato leaf roll Karnataka virus has undergone biolistic inoculation using fractional DNA dimers cloned from monopartite begomoviruses. It served as the initial evidence in 2002 (Chatchawankanphanich and Maxwell, 2002). Tomato yellow leaf curl Sardinia virus (TYLCSV) and TYLCV-[Cu] (a TYLCV strain from Cuba) were used in the first documented biolistic inoculation of TYLCV in 2003 (Ramos et al., 2003). TYLCSV clones were contagious following biolistic vaccination (for unclear reasons), but neither virus was contagious following agroinoculation vaccination (Ramos et al., 2003). Finally, plants with DNA in dimer form were cloned from the TYLCV-[Alm] (Almeria isolate), TYLCV-Mld (mild strain), and TYLCV (Morilla et al., 2005).

Examination of transgenic plants for TYLCV resistance involving *in vivo* vaccination methods under open climate or non-proficient nursery conditions is troublesome because of stringent guidelines on the genetically modified organisms (Ben Tamarzizt et al., 2009). Subsequently, a controlled immunization convention should be laid out to forestall the unwanted spread of the virus into the climate, mainly while testing new virus strains or recombinants. Two past reports portrayed the improvement of a virus vaccination framework reasonable for *in vitro* plants (Russo and Slack, 1998; Mazier et al., 2004; Al Abdallat et al., 2010). Utilizing the described system, *in vitro*-developed plants can be effectively inoculated utilizing mechanical procedures. The rule of the new vaccination technique is to submerge the foundation of the plant in an answer containing agrobacterium with an irresistible TYLCV clone. Al Abdallat et al. (2010) fostered a novel and proficient technique for *in vitro* immunization of tomato plants with TYLCV. This technique has been successfully used to uncover TYLCV opposition in wild tomatoes and permit stockpiling and spread of contaminated tomato plants under appropriately controlled conditions. Starting screening of transgenic plants with further developed protection from TYLCV utilizing the portrayed *in vitro* method is suggested

## 4 Natural resources resistant to the TYLCV in tomato

Natural TYLCV-resistant germplasm resources have been studied and characterized in numerous tomato lines, genotypes, and cultivars over the last few decades, most of which are addressed in **Table 1**. *S. pimpinellifolium*, *S. peruvianum*, *S. chilense*, *S. habrochaites*, and *S. cheesmaniae* are wild tomato that have been the focus of plant breeders’ efforts to uncover natural sources of virus resistance (Yan et al., 2018). *Ty-1*’s initial source was LA1969, (Verlaan et al., 2011; Verlaan et al., 2013) and *Ty-2* from *S. habrochaites* f. glabratum accession “B6013” (Yang et al., 2014). The self-incompatible and heterogeneous *S. chilense* wild tomato variety produces numerous alleles of the same gene in a single accession (Bai et al., 2004). LA1932 demonstrates that resistance allele 35 exists for *Ty-1*/*Ty-3* and *Ty-4* (Ji et al., 2009b). In LA2779, *Ty-3* and *Ty-6* were also discovered (Hutton and Scott, 2013). The potential that previous *S. chilense*-derived lines carry various resistance genes for TYLCV resistance in accordance with the selection process and heterogeneity of *S. chilense* (Caro et al., 2015). Finally, several tomato varieties and cultivars have recently been introduced around the world through various breeding programs, such as Yarkiy (Rumyanets), Malinovyi (Slon), and Nicola in Kazakhstan (Pozharskiy et al., 2022). Quamruzzaman et al. (2021) evaluated 75 tomato entries in Bangladesh for resistance to TYLCV infection, and 47 showed zero percent infection. Furthermore, in Egypt, two new tomato lines (TYG-1-3 and KIS-N-2-1) were resistant to TYLCV infection (Elmorsy et al., 2021). Hussain et al. (2022) screened 24 lines in Pakistan for TYLCV using disease scoring and TAS-ELISA; seven accessions, Acc-17890, AVR-261, CLN-312, AVR-321, EUR-333, CLN-352, and CLN-362, expressed resistance to TYLCV.

**Table 1:** Tomato resources resistance to TYLCV.

Genotype/Lines/Cultivars	Material Source	Resistance Gene	Notes	References
LA1969	*S. chilense*	*Ty-1*	Source of resistance	(Zakay et al., 1991; Laterrot, 1993; Zamir et al. 1994)
LA1932, LA1938, LA1960, and LA1971	*S. chilense*	*Ty-1/Ty-3*	Resistance	(Yan et al., 2018)
LA1961	*S. chilense*	*Ty-1*or *Ty-4* or *Ty-6*	Resistance	(Scott and Schuster, 1991)
LA 1968	*S. chilense*	*-*	Resistance	(Scott and Schuster, 1991)
TY52	*S. lycopersicum*	*Ty-1*	Resistance	(Wang et al., 2018b)
LA3473	*S. chilense*	*Ty-1*	Resistance	(Prasanna et al., 2015)
LA1932	*S. chilense*	*Ty-1*	Resistance	(Ji et al., 2007a)
CLN2513, CLN2514, and CLN2515	*S. lycopersicum*	*Ty-1*	Contain combined resistance derived from *Ty-1* and *Ty-2*.	(Yan et al., 2018)
BL982		*Ty-1*	Resistance	AVRDC (2002) (Yan et al., 2018)
B6013	*S. habrochaites*	*Ty-2*	Resistance	(Hanson et al., 2000; Hanson et al., 2006)
CLN2777A	*S. lycopersicum*	*Ty-2*	Resistance	(Wang et al., 2018b)
CLN2585D	*S. habrochaites*	*Ty-2*	Resistance	(Prasanna et al., 2015)
CLN2513, CLN2514, and CLN2515		*Ty-2*	Resistance	(Yan et al., 2018)
CLN2116	*S. lycopersicum*	*Ty-2*	Resistance	AVRDC (2002) (Yan et al., 2018)
LN2460G, CLN2460H, CLN2460I, CLN2460J, CLN2463O, and CLN2463P	*S. lycopersicum*	*Ty-2*	Resistance	(Yan et al., 2018)
LA2779/LA1938 LA4440	*S. chilense*	*Ty-3*	Resistance	(Ji et al., 2007a)
LA1932	*S. chilense*	*Ty-3a*	Resistance	(Ji et al., 2007a)
CA4 and GC171	*S. chilense*	*Ty-3*	Resistance	(Prasanna et al., 2015)
LA1969	*S. chilense*	*Ty-3b*	Resistance	(Ji et al., 2007a)
LA1932	*S. chilense*	*Ty-4*	-less efective against TYLCV. -Increase resistance levels in combination with *Ty-3*	(Ji et al., 2009b; Kadirvel et al., 2013)
GC171 LA4440	*S. chilense*	*Ty-4*	Resistance	(Prasanna et al., 2015; Lee et al., 2020)
Tyking	*S. lycopersicum*	*ty-5*	Recessive resistance	(Lapidot et al., 2015)
LA1938	*S. peruvianum*	*ty-5*	Recessive resistance	(Anbinder et al., 2009; Hutton et al., 2012)
TY172	*S. peruvianum*	*ty-5*	Recessive resistance	(Friedmann et al., 1998; Anbinder et al., 2009)
AVTO1227	*S. lycopersicum*	*ty-5*	Recessive resistance	(Wang et al., 2018b)
Fla.8753, Fla. 344 and Fla.8062	*S. chilense*	*ty-5*	high level of resistance due to the presence of *ty-5* and *Ty-6*.	(Hutton et al., 2012; Scott et al., 2015)
Fla.8624 and Fla.8638B	*S. chilense*	*Ty-6*	moderate level of resistance	(Scott et al., 2015)
LA2779	*S. chilense*	*Ty-6*	Resistance	(Scott et al., 2015)
Fla.8753, Fla.344 and Fla.8062	*S. chilense*	*Ty-6*	Introduce high level of resistance due to the presence of *ty-5* and *Ty-6*.	(Hutton et al., 2012; Scott et al., 2015)
Fla.456	*S. chilense*	*Ty-6*	Resistant	(Bian et al., 2007; Gill et al., 2019)
UPV-16910	*L. hirsutum*	–	partially tolerant	(Picó et al., 2002)
PI-126944^b^	*Lycopersicon hirsutum (L. hirsutum)*	–	Resistant	(Picó et al., 1998; Roselló and Nuez, 1999)
PI-211840	*S. pimpinellifolium*	–	Resistant	(Scott and Schuster, 1991)
LA386	*L. hirsutum*	–	Resistant	(Scott and Schuster, 1991)
PI 212408, LA373, LA1582, LA1478 and Hirsute	*S. pimpinellifolium*	–	Resistant	(Scott and Schuster, 1991)
TY-20, LA 121and EC 104395	*L. esculentum*	–	Resistant	(Scott and Schuster, 1991)
LA 1401	*L. cheesmanii*	–	Resistant	(Scott and Schuster, 1991)

## 5 Natural genes resistant to TYLCV in tomato

**Figure 4:**
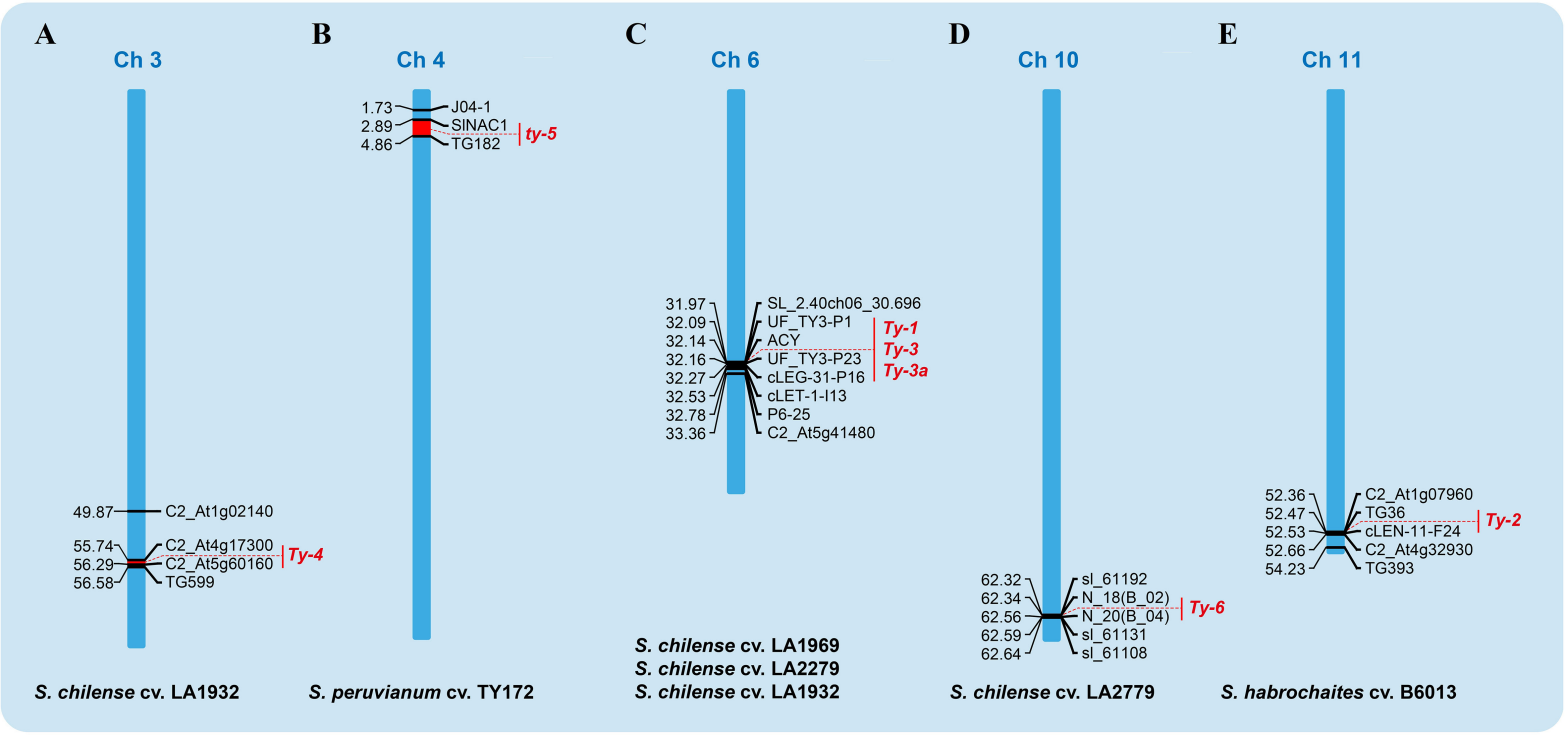
Mapping of TYLCV resistance genes on tomato chromosomes. **(A)** The site of *Ty-4* on chromosome 3 of *S. chilense* cv., **(B)** the site of *ty-5* on chromosome 4 of *S. peruvianum* cv., **(C)** the site of *Ty-1*, *Ty-3* and *Ty-3a* on chromosome 6 of *S. chilense* cv. (LA1969, LA2279 and LA1932 respectively), **(D)** the site of *Ty-6* region on chromosome 10 of *S. chilense* (LA2279) and **(E)** the *Ty-2* region on chromosome 11 of *S. habrochaites* cv.

Six distinct genes (*Ty-1*, *Ty-2*, *Ty-3*, *Ty-4*, *ty-5*, and *Ty-6*) are located on different tomato chromosomes (**Figure 4**) and provide varying levels of resistance in wild germplasm when transfected into commercial cultivars (Anbinder et al., 2009; Hutton and Scott, 2013; Prabhandakavi et al., 2021). Except for *ty-5*, which has recessive inheritance, all of these genes are dominant resistance (Ren et al., 2022). *Ty-1*, *Ty-3*, *Ty-4*, and *Ty-6* are derived from *S. chilense* (Hutton and Scott, 2013), whereas *Ty-2* and *ty-5* may be derived from *S. habrochaites* and *S. peruvianum*, respectively (Anbinder et al., 2009; Wolters et al., 2015). It is now possible to introduce resistance genes without causing cross-resistance or to pyramid numerous resistance genes in marker-assisted breeding thanks to the development of molecular biotechnology.

### 5.1 *Ty-1*

*Ty-1* was included in LA1969 from *S. chilense* (Verlaan et al., 2011; Verlaan et al., 2013). The first was identified and located on tomato chromosome 6’s short arm by Zamir et al. (1994). *Ty-1* is related to the *Mi-1* gene cluster at the REX-1 locus, indicating that *Ty-1* is derived from the short arm of chromosome 6 (Milo, 2001). *Ty-1* was discovered on the long arm of chromosome 6 and linked to the Ty-3 locus (Pérez de Castro et al., 2013). de Castro et al. (2007) found that *Ty-1* is associated with the CT21 marker, which is situated below the long arm’s centromere, in a different investigation. Also, *Ty-1* has recently been fine-mapped and cloned, and it has been shown to contain an allele of a gene coding for an RNA-dependent RNA polymerase (RDR) (Verlaan et al., 2011; Verlaan et al., 2013). The *Ty-1* gene thus represents a distinct family of genes that increase the transcriptional silence of viral genes to confer disease resistance or tolerance. Recent research has demonstrated that in genetically modified *N. benthamiana* plants, the *Ty-1* gene can also confer resistance to the beet curly top virus (a genus Curtovirus) (de Nazaré Almeida dos Reis et al., 2020; Voorburg et al., 2020). More research is needed to determine the effect of the *Ty-1* gene on ssDNA viral and subviral diseases associated with tomatoes in the Neotropics. More viruses (14 versus 6 species) were found in tomatoes lacking the *Ty-1* gene, including a gemycircularvirus (*Genomoviridae*), a new alpha-satellite, and two novel *Begomovirus* species (de Nazaré Almeida dos Reis et al., 2020; Nehra et al., 2022). A novel *Begomovirus* was found only in the *Ty-1* pool in the de Nazaré Almeida dos Reis et al. (2020) survey, and it was the only species associated with severe symptoms in *Ty-1* plants. Three ORFs were predicted to be *T-y1*/*Ty-3*, and the *Ty-1* gene has been identified as genomic alleles for genes *Ty-3*, *Ty-3a*, and *Ty-3b* (Jensen et al., 2007; Ji et al., 2007a; Verlaan et al., 2011; Verlaan et al., 2013). Though since plants with this factor allow for a minor onset of symptoms, primarily in the apical meristem region, and then gradually recover as the plant grows/develops, phenotypic expression of the *Ty-1* gene is more accurately described as a tolerance response (Cooper and Jones, 1983; de Nazaré Almeida dos Reis et al., 2020). Finally, *Ty-1* is the most common resistance gene used in tomato breeding. TYLCV, on the other hand, undermines *Ty-1* resistance when co-infected with a betasatellite. This suggests that the TYLCV/betasatellite complex can bypass the commonly used *Ty-1* resistance gene (Ren et al., 2022).

### 5.2 *Ty-2*

Hanson et al. (2000) were the first to report the presence of a resistance introgression (*Ty-2*) derived from *S. habrochaites* accession B6013 in the tomato-resistant line H24, which was developed from *S. habrochiates* f. glabratum accession ‘B6013’ (Kalloo and Banerjee, 1990) and contains an introgression spanning at least 19 cM from TG36 (map position 84 cM) to TG393 (103 cM) (Ji et al., 2009a). According to Ji et al., the *Ty-2* gene was restricted in a 500kb introgressed region between markers C2 At2g28250 (physical location 51.307Mb) and T0302 (51.878 Mb) (2009). Later, Yang et al. (2014) reduced the *Ty-2* region to a 300 kb distance between markers UP8 (51.344 Mb) and M1 (51.645 Mb) at the end of chromosome 11’s long arm (Barbieri et al., 2010). *Ty-2* is one of the essential TYLCV resistance genes used in tomato breeding, but it is ineffective against many TYLCV strains worldwide (Shen et al., 2020). Numerous tomato breeding projects have attempted to identify recombinants containing *Ty-2* and *I-2*, but have so far been unsuccessful. *Ty-2* is intimately associated with susceptibility to Fusarium wilt race 2, and efforts to date have been ineffective (Ji et al., 2007a). There have been numerous attempts to define the gene structure and produce a perfect map of the *Ty-2* locus. However, there is no concrete proof that a particular gene is responsible for *Ty-2*-mediated resistance (Yamaguchi et al., 2018).

### 5.3 *Ty-3*

*Ty-3* was identified in *S. chilense* accessions such as LA1932, LA1938, and LA2779, and it was first located on the long arm of chromosome 6 in these accessions (Ji et al., 2007a). Resistance to the TYLCV and begomovirus tomato mottle virus (ToMoV) is possible (Ji et al., 2007a). *Ty-3* and *Ty-1* areas overlap, indicating the potential for alleles (Verlaan et al., 2013). *Ty-1* and *Ty-3* have been focused breeding efforts that have been joined to create trade hybrids around the globe. The *Ty-1*/*Ty-3* gene from *chilense* (LA1969) encodes an RNA-dependent RNA polymerase that participates in antiviral RNA silencing and was the first and only TYLCV dominant resistance gene to be cloned. *Ty-1* and *Ty-3* are RDR type homologs of *A. thaliana RDR3*, *RDR4*, and *RDR5* genes, which have yet to be assigned functions (Verlaan et al., 2013).

Increased amounts of TYLCV-specific siRNA targeting the V1 promoter area were seen in plants with *Ty-1*/*Ty-3*, along with cytosine methylation in the region’s promoter, which suggested an increased transcriptional gene silencing (TGS) resistance mechanism (Butterbach et al., 2014). In *Solanum* species, the catalytic region of the *Ty-1*/*Ty-3* gene is conserved *S. chilense*, and six other wild species of *Solanum* have a 12-base pair introduction in *Ty-1*/*Ty-3*, albeit it is not entirely linked to TYLCV resistance. SNPs targeting resistant *Ty-1*/*Ty-3* alleles can improve allele-specific markers (Caro et al., 2015). However, the co-dominant SCAR marker P6-25 was utilized to identify the *Ty-3*, *Ty-3a*, and *Ty3b* alleles in three Chinese accessions, LA2779, LA1932, and LA1969, at a distance of 25 cM. Tomato with begamovirus resistance (Ji et al., 2007a; Ji et al., 2007b).

### 5.4 *Ty-4*

*Ty-4* originated from *S. chilense* accession LA1932; it has been located on the third chromosome’s long arm (Ji et al., 2009b; Dhaliwal et al., 2020). Only 15.7% of the entire variation was accounted for by *Ty-4*, which had no impact on TYLCV resistance; in contrast, 59.6% of the variation was accounted for by *Ty-3*, which originated from *S. chilense* (Ji et al., 2009b). Although we did not test for bipartite begomovirus, *Ty-4* is efficient against TYLCV. However, in Guatemala, inbred lines carrying both *Ty-3* and *Ty-4* were more resistant than lines carrying just *Ty-3* (D.P. Maxwell, unpublished data), showing that *Ty-4* is resistant to a number of (up to 7) bipartite begomoviruses that are active, and these viruses are likely prevalent there (Nakhla et al., 2005). Compared to other *Ty* genes, *Ty-4* is less efficient against TYLCV (Kadirvel et al., 2013).

### 5.5 *ty-5*

The *ty-5* has been identified in the tomato reproducing line TY172, which is descended from four different elevations of *S. peruvianum* (PI126926, PI126930, PI390681, and LA0441) (Friedmann et al., 1998; Hutton et al., 2012; Lapidot et al., 2015). The genetic analysis has shown that *ty-5*-mediated blockage on chromosome 4 is constrained by a substantial quantitative trait loci (QTL) (Anbinder et al., 2009). The *ty-5* co-isolates with the marker SlNAC1 and is acquired latently (Anbinder et al., 2009; Hutton et al., 2012). It should be merged into the two guardians of a crossover, according to *ty-5* features. However, the ability to apply related markers will consider a skillful fusion of this allele into cutting-edge material by marker-assisted selection. The *ty-5* confers broad-spectrum resistance to geminiviruses and was effective against two representative begomoviruses in China, TYLCCNV/TYLCCNB and TbLCYnV. *ty-5* also provided partial resistance to BCTV, a virus in the Curtovirus genus. Subsequently, *ty-5* was resistant to TYLCV co-infected with a betasatellite (Ren et al., 2022).According to Levin et al. (2013) cloning study, the *ty-5* gene codes for a pelota homolog related to protein translation. TYLCV opposition is linked to a T-to-G transversion in the pelota allele’s coding region. The *ty-5* gene demonstrates that the Pelota gene is a TYLCV host susceptibility factor by addressing the functionality of the *Pelota* gene’s allele (Lapidot et al., 2015). Loss-of-work mutations have demonstrated resistance to various Gemini viruses at the Pelo homologous locus (Yan et al., 2021).

### 5.6 *Ty-6*

The *Ty-6* is generated from *S. chilense* accession LA2779 and is a newly discovered TYLCV resistance locus (Hutton and Scott, 2014; Scott et al., 2015; Gill et al., 2019). According to preliminary mapping data, it is located in a region of about 3 Mb on the long arm of chromosome 10 (Gill et al., 2019). A notable discovery was the identification of *Ty-6*, which expands breeders’ toolkit of *Ty* genes and confers resistance to bipartite and begomoviruses monopartite (Prasad et al., 2020; Gupta et al., 2021). Contrary to popular belief, *Ty-6* is primarily responsible for ToMoV resistance in lines such as Fla. 8680, not *Ty-3* (Scott et al., 2015; Gill et al., 2019).

Similarly, even though *ty-5* and *Ty-6* work together to cause TYLCV in lines like Fla. 8638B and Fla. 8472, Gill et al. (2019) discovered that *ty-5* is ineffective against ToMoV and that the existence of *Ty-6* in such lines is what causes the bipartite resistance. Given that *S. chilense* is in the pedigree of all *Ty-6*-containing UF/IFAS lines evaluated thus far, Scott et al. (2015)‘s claim that this species was the source of *Ty-6* in Fla. 8624 and Fla. 8638B is probably accurate. *Ty-6* will probably be very helpful for many tomato breeding efforts worldwide due to its wide efficacy against mono- and bipartite begomoviruses and the complementing resistance it provides when combined with other genes (Gill et al., 2019). Despite discovering numerous SNPs associated with *Ty-6* that can be used for breeding, none were consistently polymorphic between *Ty-6* and *Ty-6* breeding lines (Gill et al., 2019).

## 6 Molecular markers for resistance to TYLCV in tomato

Indirect selection of desirable plant phenotypes employing linked molecular DNA markers as a binding mechanism is known as marker-assisted selection (MAS) (El-Sappah et al., 2019; El-Sappah and Rather, 2022). According to the MAS theory, a gene of interest is present when a closely connected marker is found (Jiang, 2013). The development of novel resistant crops has numerous advantages. The two main benefits of molecular breeding are that it takes less time (Xu and Crouch, 2008) and is less expensive than field screening (Morris et al., 2003). Additionally, it is less damaging to the environment than pesticides (Afify et al., 2022). The tomato is one of the most remarkable plants for commercial breeding with molecular markers (Hanson et al., 2016). Molecular markers for MAS in CAPS and SCAR markers have been used to develop the *Ty-1*/*Ty-3* resistance gene (Ji et al., 2009a; Nevame et al., 2018; Kim et al., 2020). Although, concerns have been voiced over their physical proximity to the resistance genes in the genome, posing the possibility of false-positive or false-negative outcomes in breeding programs (Antignus, 2007; Yang et al., 2014; Nevame et al., 2018). Gene-specific marker technology was developed to create gene-specific molecular markers to get around the issues mentioned earlier (Ramkumar et al., 2010; Ramkumar et al., 2011). As a result, functional markers, resistance gene-based markers (RGM), gene-targeted markers, and RNA-based markers have all been developed (Sorri et al., 1999; Kasai et al., 2000; Valkonen et al., 2008). Functional markers are polymorphic DNA sequences that play a role in phenotypic trait variation, according to Andersen and Lübberstedt (2003), whereas gene-targeted markers are gene-specific and can mark untranslated regions (Arnholdt-Schmitt, 2005; Varshney et al., 2007). RGM can make it possible to detect resistance genes in fresh germplasm and isolate populations to support plant gene pyramids (Poczai et al., 2013). The DNA markers for these TYLCV resistance genes are presented in **Table 2**. Since *Ty-1* and *Ty-3* were discovered separately from various tomato germplasms, various DNA markers closely related to *Ty-1* or *Ty-3* have been applied in tomato breeding projects. However, new research has revealed that these two are allelic-related at a single locus (Verlaan et al., 2013; Caro et al., 2015). Research is being done on the resistance levels and spectra that *Ty-1* and *Ty-3* bestow. *Ty-2* resistance was successfully selected using the SCAR marker T0302 on chromosome 11, but it has recently been reported in a 300 kb region, and efforts are being made to identify its genes in order to create gene-based markers (Wolters et al., 2015). Based on the information provided by Lapidot et al. (2015), a gene-based dCAPS marker for *ty-5* was developed.

**Table 2:** Genetic markers assisted breeding to TYLCV resistance.

Marker	Gene	Marker Type (restriction Enzyme)	Forward/Reverse of Marker sequences	Product Size (bp)	References
SCAR1	*Ty-1*	SCAR	5'-CAATTTATAGGTGTTTTTGGGACATC-3' 5'-GTTCAACACTTGGCCAATGCTTACG-3'	R:350 S:610	(Nevame et al., 2018)
JB1	*Ty-1*	SCAR	5'-AACCATTATCCGGTTCACTC-3' 5'-TTTCCATTCCTTGTTTCTCTG-3'	R: 450 S: 400	(de Castro et al., 2007)
TG178	*Ty-1*	SCAR	5'-GAGTCCCTAACGAATGGTCCTACT-3' 5'-GCAGACAAATGCTCAAAGGTCACACC-3'	Multiple bands	(Barbieri et al., 2010)
Ty1-TaqI	*Ty-1*	CAPS (TaqI)	5'-ATGAAGACAAAAACTGCTTC-3' 5'-TCAGGGTTTCACTTCTATGAAT-3'		(Jung et al., 2015)
Ty1-SspIHJ	*Ty-1*	SNP	5'-GGTTGGTCTCCTTGATAGTCATGT-3' 5'-TCCACTTGAAGCTTAATAGTCTTTGA-3'	R:118	(Jung et al., 2015)
Ty1-3	*Ty-1,Ty-3*	InDel	5'-GGGTGATCCGTTGATTGAAG-3' 5'-TCTTCTTGATAGGACGACGTGA-3'		(Hu et al., 2014)
14IY218	*Ty-1/3*	CAPS (SspI)	5'-ATGAAGACAAAAACT GCT TC-3' 5'-TCAGGG TTTCACTTCTATGAA T-3'	R: 383, 226 S: 609	(Jung et al., 2015)
M2	*Ty-1/3*	SCAR	5'-GATCCGTTGATTGAAGAAAT-3' 5'-AGGAAGAGGAGAGACAATCC-3'	R: 264 S: 252	(Jung et al., 2015)
TY-1/3_K	*Ty-1/3*	SCAR	5'-ACAGGAAAAATGGGTGATCC-3' 5'-CCTGCTCCTTGCAGATTCTA-3'	R: 114 S: 102	(Chen et al., 2015)
Ty1-SspI	*Ty1/3*	CAPS (SspI)	5'-ATGAAGACAAAAACTGCTTC-3' 5' -TCAGGGTTTCACTTCTATGAAT-3'	R: 608	(Jung et al., 2015)
Ty1-BglII	*Ty1/3*	CAPS (BglII)	5' -ATGAAGACAAAAACTGCTTC-3' 5' -TCAGGGTTTCACTTCTATGAAT-3'		(Jung et al., 2015)
ACY	*Ty-1,Ty-3, Ty-3a*	Indel	5' -GAAGCACAGATTGAAGAAAACC-3' 5' -CCTTATGATGTCTCGTGAAAGG-3'	R: 132 S:123	(Nevame et al., 2018)
P1-16	*Ty-2*	SCAR	5' -CACACATATCCTCTATCCTATTAGCTG-3' 5'-CGGAGCTGAATTGTATAAACACG-3'		(Yang et al., 2014)
SCAR2 (T0302)	*Ty-2*	SCAR	5'-TGGCTCATCCTGAAGCTGATAGCGC-3' 5'-AGTGTACATCCTTGCCATTGACT-3'	R:900 S:800 or 791	(Garcia et al., 2007; Nevame et al., 2018)
20IY10	*Ty-2*	InDel	5'-GTTCTATCACAAGACTTGCCA-3' 5'-TGCATTCACCATTGATGTATAAGA-3'	R: 738 S: 600	(Lee et al., 2020)
TES0344	*Ty-2*	SSR	5'-GCCTTTTCCCACTTATATTCCTCTC-3' 5'-ACACATACGACGTTCCGTCA-3'	R: 190 S: 205	(Yang et al., 2012)
Ty2-UpInDe	*Ty-2*	InDel	5'-ACCCCAAAAACATTTCTGAAATCCT-3' 5'-TGGCTATTTTGTGAAAATTCTCACT-3'	R:120 S:213	(Kim et al., 2020)
Ty3-InDel4	*Ty-3*	CAPS (BstZ17I)	5'-CCTATCCTCAGTGTTTCGGTCA-3' 5'-GGCGAAAGACTTTGTGTACACA-3'	R: 353/325 (678) S: 669 (669)	(Kim et al., 2020)
Ty3- SNP9	*Ty-3*	CAPS (MfeI)	5'-CCTATCCTCAGTGTTTCGGTCA-3' 5'-GGCGAAAGACTTTGTGTACACA-3'	R:678 (678) S: 555/114 (669)	(Kim et al., 2020)
Ty3-SNP17	*Ty-3*	CAPS (RsaI)	5'-TCTCAGGTGATGCTGAGCAC-3' 5'-AGAGAACGAAAACGAAATTTCAAACA-3'	R:497/148/65/52/51 (813) S:562/148/52/51 (813)	(Kim et al., 2020)
P6-25	*Ty-3* *Ty-3a* *Ty-3b*	SCAR	5'-GGTAGTGGAAATGATGCTGCTC-3' 5'-GCTCTGCCTATTGTCCCATATATAACC-3'	R: 623bp for *Ty-3a* 453bp for *Ty-3 *and 660 bp for *Ty-3b* S: 320bp for ty-3	(Ji et al., 2007a; Salus et al., 2007; Nevame et al., 2018)
Cauty4	*Ty-4*	InDel	5'-GGGCAACTCAATGGTGAAAC-3' 5'-TCTGAATGTAGGGCCAAAGG-3'		(Hu et al., 2014)
18IY23	*Ty-4*	dCAPS (StuI)	5'-AGAAGAAATCCAAGAAAAGCAATA AGA ATGAGG CC-3 ' 5'-CTT GTAATCACG TCCACAACG-3'	R: 304 S: 269, 35	(Lee et al., 2020)
18IY13	*Ty-4*	InDel	5'-CTTCTGTTCTATGCAGGTGTG-3' 5'-GGATACAACTGTCAACGCAC-3'	R: 228 S: 200	(Lee et al., 2020)
ty-5	*ty-5*	SSR	5'-GACTGCATTGGATTTGGCTT-3' 5'-CAATCGATGCACAAAACACC-3'		Yang et al. (2016)
14IY5	*ty-5*	dCAPS (RsaI)	5'-TTCAAGTCCTTCTTCAACATAGATTTA AACAACAATTATAGA-3' 5'-GATAAAAAAGTTACCTGT-3'	R: 300 S: 260, 40	(Lee et al., 2020)
AVRDC-TM719	*ty-5*	SSR	5'-TCGATTTGGAATGAGTTTTC-3' 5'-TGAAATAGATTTGTCAGGTGTT-3'	S: 237	(Chen et al., 2015)
SLM4-34	*ty-5*	SSR	5'-GACCATTAACCTCGATCA-3' 5'-GAAAGTCATGTGAATAGCAG-3'	Multiple bands	(Kadirvel et al., 2013)
SINAC1 (TAQ I)	*ty-5*	SSR	5'-TGCCTGGTTTCTGCTGTCA-3' 5'-TAAAGCTGAAGAAGGACTTACCCT-3'	Multiple bands	(Anbinder et al., 2009)
AVRDC-TM273	*ty-5*	SSR	5'-GGTGCTCATGGATAGCTTAC-3' 5'-CTATATAGGCGATAGCACCAC-3'	R: ~180 S: 173	(Chen et al., 2015)
AVRDC-TM81	*ty-5*	SSR	5'-GTATGGAGAGTCGAGTCCTG-3' 5'-CCATGATAAGTAGCGAGAGG-3'	S: 153	(Chen et al., 2015)
AVRDC-TM70	*ty-5*	SSR	5'-TTTCTTTGTTTCCTTTCAGTG-3' 5'-GCCTTGGACAAGGTACAATA-3'	Multiple bands	(Chen et al., 2015)
AVRDC-TM947	*ty-5*	SSR	5'-TGCGTCTAGTTTTCTTTGTTT-3' 5'-CAAGCTGAAAGGAATTCAAC-3'	Multiple bands	(Chen et al., 2015)

R and S at product size mean Resistance and Susceptible, respectively.

## 7 Mechanism of natural resistance to the TYLCV in tomato

**Figure 5:**
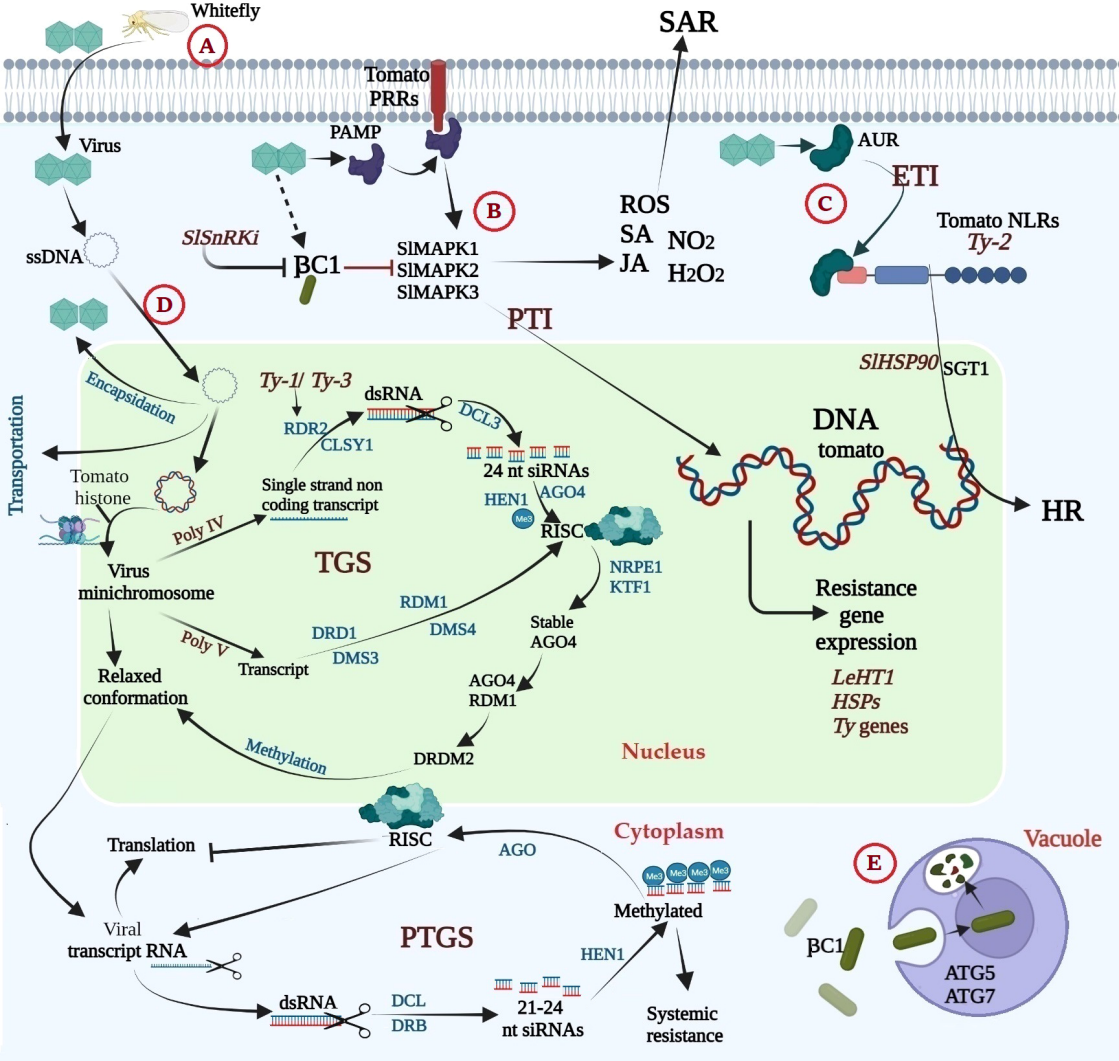
Molecular response of tomato during TYLCV infection; **(A)** the whitefly carries the virus and transfer it to the tomato during its feeding, **(B)** the tomato’s first line of defense is recognition of pathogen-associated molecular patterns (PAMP) by host pattern recognition receptors (PRRs), resulting in activation of PAMP triggered immunity (PTI), **(C)** the tomato second line of defense, plants have evolved cytoplasmic R proteins (nucleotide binding–leucine-rich repeat proteins, NB-LRR) (NLRs) i.e., *Ty-2* gene that recognizes the presence or activity of specific virus effectors like avirulence AVR, resulting in effector triggered immunity (ETI), **(D)** Once the viral ssDNA is released from the capsid, it enters the cytoplasm of the infected cell and subsequently enters the cell nucleus, where it undergoes rolling-circle and recombination dependent replication and plant immunity begin by inducing both TGS and PTGS, with the help of *Ty-1*/*Ty-3* genes, **(E)** Tomato autophagy, where Rep protein of TLCYnV, CLCuMuB βC1 protein interacts with autophagy related protein NbATG8 through its ATG8 interacting motif (LVSTKSPSLIK) and directs it for degradation. This figure was made using BioRender.

The tomato plant’s molecular and cellular responses to TYLCV infection are depicted in **Figure 5**. Six partially overlapping open reading frames are present in the single-stranded, circular, bidirectionally structured DNA genome of TYLCV (Gronenborn, 2007). Due to their restricted coding capability, like most viruses, they depend on the host cell’s machinery and their proteins for the infection cycle (Hanley-Bowdoin et al., 2004). Viral ssDNA exits the capsid and moves into the cytoplasm and nucleus of infected cells, where it engages in a rolling cycle and recombination-dependent replication (Gutierrez, 1999). The freshly replicated viral ssDNA can be transformed into dsDNA, which can be used as a template for further replication or transcription. It can also be encapsulated by viral activity proteins for transport *via* plasmodesmata from infected cells to nearby cells or packaged in a contagious kind to allow for long-distance viral transmission (Gutierrez, 1999; Hanley-Bowdoin et al., 2013). In addition, geminiviruses rely extensively on host proteins to complete their infection cycle because they have little capacity for coding. To control cell division and the cell cycle, coordinate with multiple cellular mechanisms, and affect host components at various cellular levels, their replication, and transcriptional processes depend on host enzymes (Hanley-Bowdoin et al., 2013). Additionally, they produce short RNAs and block numerous TGS and post-transcriptional gene silencing (PTGS) components by encoding a variety of proteins that disrupt PTGS pathways (Raja et al., 2010; Hanley-Bowdoin et al., 2013; Gnanasekaran et al., 2019). In order to reduce symptoms in different crops and viruses, these proteins also utilize viral suppressors of the RNA silencing mechanism (VSRs) (Ceniceros-Ojeda et al., 2016; Basu et al., 2018). Geminivirus VSRs are versatile proteins that support the viral life cycle and weaken host defense (Teixeira et al., 2021). They can abolish PTGS and TGS in any of the three stages of the operation, and they can also directly or indirectly impact DNA methylation through events that happen after TGS (Loriato et al., 2020). Mechanically, RNA silencing machinery components are either actively inhibited or prevented from accumulating (expression) by geminivirus VSRs. Representatives of the viral repertoire known as C4/AC4 interact with and sequester dsRNA precursors from DCL cleavage and siRNAs from RISC loading in order to prevent antiviral RNA silencing. AC1 (bipartite geminiviruses) and C1 (monopartite geminiviruses) have been shown to function as powerful VSRs in both PTGS and TGS (Amin et al., 2011; Sunitha et al., 2013). Additionally, during the amplification phase, a subsequent event of TGS, AV2 and V2 impair host methylation activity and block antiviral RNA silencing (Luna et al., 2017; Wang et al., 2018a; Luna et al., 2020). Through a number of methods, the C1 protein encoded by the -satellite genome functions as an effective VSRs, preventing the methylation of viral genomes in plants and PTGS that have been infected (Yang et al., 2011; Saeed et al., 2015). To identify and trigger defense reactions against pathogens, the plant immune system has created a multi-layered receptor system. The initial line of defense, according to the traditional zig-zag model of plant immunity, is the recognition of pathogen-associated molecular patterns (PAMPs) by host pattern recognition receptors (PRRs), which activate PAMP-triggered immunity (PTI) (Jones and Dangl, 2006). Successful pathogens release effectors in response, suppressing the PTI response and causing effector-induced susceptibility (ETS). Receptor-like kinases (RLKs) and receptor-like proteins, two transmembrane receptors, are responsible for the appearance of PRRs. These PRRs realize damage-associated molecular patterns (DAMPs), which are exclusively expressed by endogenous danger signals supplied by pathogens or host plants during infection (Macho and Zipfel, 2015). To create an active immunological complex, RLKs and RLPs typically need a co-receptor (Ma et al., 2016). PAMPs and DAMPs function as bonds to enhance the dimerization and oligomerization of PRRs, one-way transmembrane receptors interacting with RLK co-receptors to initiate signaling and activate immune complexes (Macho and Zipfel, 2015). Following PTI activation, the MAP kinase cascade is activated, PTI-related defense genes are induced, ethylene and salicylic acid are synthesized, and callose is deposited (Teixeira et al., 2021; Raza et al., 2022). PTIs appear to be a component of the host’s arsenal of defense against geminivirus infection, despite the fact that geminivirus PAMPs and their associated PRRs have not been described. The TYLCCNB-C1 protein interacts with and is phosphorylated by tomato sucrose non-fermentation 1-associated kinase (*SlSnRK1*), which may cause proteasomal destruction (Shen et al., 2011). It has been demonstrated that the TYLCCNB-C1 protein inhibits methylation-mediated RNA silencing and its function in lowering PTI in tomato plants. Another line of defense is available. Where plants produce cytoplasmic R proteins, also known as NB-LRRs (nucleotide-binding leucine-rich repeat proteins), which can detect the presence or absence of specific viral impacts, such as avirulent (AVR) activity and result in effector-induced immunity (ETI) (Jones and Dangl, 2006). According to their N-terminal structures, plant NLRs are divided into two groups: CC-NLRs (CNLs) and Toll/Interleukin-1 (TIR)-NLRs (TNLs) (Selth et al., 2004). *Ty-2* is a CC-NBS-LRR (CNL) type gene member of the CNL genes with an I-2-like subclass (Shen et al., 2020). When *Ty-2* is co-expressed and activated with the TYLCV Rep/C1 protein, a hypersensitive responses (HR) response is produced.

ETIs frequently cause HR and systemic acquired resistance (SAR) (Saile et al., 2020). Recent research suggests that there may not be a very distinct difference between PAMPs and effectors or between PAMP receptors and resistance-causing proteins (Thomma et al., 2011). As a result, PTI and ETI are not always different defensive reactions; instead, both defensive reactions can be strong or weak depending on the contact circumstances. Thus, detecting danger signals, whether they come directly from microorganisms (PAMPs and effectors) or through damage to or change of eukaryotic host structures, can recapitulate the activation of innate plant defense. The geminivirus AC2/C2 protein, a viral effector required for productive infection and can occasionally cause HR, seems to fit these criteria (Roy, 2016).

In general, plant defense responses triggered by direct or indirect effector sensing by NLR genes involve a variety of downstream signaling pathways, including phytohormones involved in defense, MAPK signaling cascades, and a set of defense-related genes (e.g., *WRKY* transcription factors) (Elmore et al., 2011; de Ronde et al., 2014; Boualem et al., 2016; Rasheed et al., 2022). After TYLCV inoculation, the *SlMAPK1*, *SlMAPK2*, and *SlMAPK3* were differently upregulated and activated (Li et al., 2017; Guo et al., 2021). Rapid reactive oxygen species (ROS) burst and activation of MPK3/MPK6 are two distinct early signaling events in the plant immune system (Xu et al., 2014; Edris et al., 2021; Elebeedy et al., 2022; Sattar et al., 2022). The SA synthesis can result from H_2_O_2_ buildup (León et al., 1995); however, TYLCV also promotes SA accumulation early in infection (Morinaka et al., 2006). High SA and H_2_O_2_ can activate the *PR* genes expression locally (Peleg-Grossman et al., 2010).

In order to silence the expression of viral genes, geminiviruses must contend with plants on two major defenses. PTGS damages viral mRNA, while methylation-mediated TGS targets viral minichromosomes (Gupta et al., 2021). Geminivirus DNA enters the nucleus after combining with coat proteins and then attaching to the host’s histone proteins; ssDNA is replicated in the nucleus in double-stranded form and exists as minichromosomes (Abouzid et al., 1988; Gupta et al., 2021). The RNA-directed DNA methylation device (RdDM) employs transcriptional gene silencing to silence viral gene expression and reduce viral minichromosomes by taking advantage of the plant’s response to the invasion (TGS) (Vanitharani et al., 2005; Zarreen and Chakraborty, 2020).Virus-produced Plant cytoplasmic siRNA-mediated silencing pathways specifically target RNA transcripts (Gupta et al., 2021). The PTGS pathway is essential for host genes’ expression, development, and defense (Chen et al., 2004).

RDR2 participates in tomatoes’ TGS pathway and antiviral defense (Mourrain et al., 2000; Xie et al., 2001; Qi et al., 2009). Element members of the *Ty-3*/Gypsy-like superfamily of retrotransposons, which are transcriptionally repressed through the RdDM pathway, are upregulated due to the loss of RDR2 function (Jia et al., 2009). The TYLCV virus has two wild-type tomato resistance alleles, *Ty-1* and *Ty-3*. These alleles are members of the RDR lineage and encode the DFDGD motif (Verlaan et al., 2013; Caro et al., 2015). Other research has demonstrated that *Ty-1* increases antiviral RNAi responses, which is implied by elevated vsiRNA levels and elevated cytosine methylation in the viral DNA genome in tomatoes treated with *Ty-1* (Butterbach et al., 2014). The viral genome’s cytosine methylation and RNA silencing are hypothesized to be regulated by several *Ty* genes (Verlaan et al., 2013; Butterbach et al., 2014; Caro et al., 2015).

Finally, autophagy, a conserved evolutionary process that recycles damaged or unneeded cellular components within cells, is another method of plant defense against TYLCV (Haxim et al., 2017; Yang et al., 2019a). According to studies on plant DNA and RNA viruses, autophagy has a potential antiviral role in host innate and adaptive immunity (Hafrén et al., 2017; Haxim et al., 2017; Gupta et al., 2021). The TLCYnV Rep protein CLCuMuB C1 interacts with the autophagy-related protein NbATG8 and regulates its degradation *via* the ATG8 interaction motif (LVSTKSPSLIK) (Gupta et al., 2021). More research, particularly in tomatoes, is needed to understand how autophagy is regulated during viral infection and determine whether blocking the proviral autophagy pathway could prevent diseases.

## 8 Challenge and prospects

The TYLCV may have originated from seeds because viral particles can stay in the seed after infection and pass on to the following generation (Albrechtsen, 2006; Baldodiya et al., 2020). Strict quarantine laws, integrated pest management, and traditional breeding are only a few methods to stop the spread of TYLCV (Prasad et al., 2020). TYLCV transmission is one of several recent transgenic strategies and traditional methods used to combat virus transmission (Pramanik et al., 2021; Asseri et al., 2022). Tomato plants that express TYLCV gene segments, such as replication-associated protein (Rep) (Antignus et al., 2004) or capsid protein (CP) provide resistance to the virus (Yang et al., 2004). Another study found that the model plant *N. benthamiana* overexpressed recombinant antibodies directed against the Rep protein and displayed decreased TYLCV symptoms (Safarnejad et al., 2009; Reyes et al., 2013). Immunization against TYLCV was successfully developed using viral gene silencing *via* RNAi-mediated techniques (Ammara et al., 2015; Leibman et al., 2015; Fuentes et al., 2016); for example, viral resistance was demonstrated by silencing the tomato *SlPelo* gene (Lapidot et al., 2015). Meanwhile, the overexpression of plant immunity-related genes immunity is a reasonable strategy for increasing pathogen tolerance in plants. For instance, *SlMAPK3* or *SlLNR* overexpression reduces TYLCV pathogenicity in tomato plants (Li et al., 2017; Yang et al., 2019b). Furthermore, *SlGRXC6* overexpression promoted plant growth, inhibited viral infection, and delayed TYLCV symptom development (Zhao et al., 2021).

Traditional breeding and transgenic approaches to TYLCV infection control are generally promising, but they have several drawbacks. Besides, they have been around for a long time and faces the risk of losing essential characteristics due to traditional breeding domestication (Jansson et al., 2017; Migicovsky and Myles, 2017). The main disadvantage of transgenic techniques is that the transgene must be expressed steadily to achieve a pathogen-tolerant phenotype. Thus the organisms must be classified as genetically modified (GMOs) (Shelake et al., 2019). Plant viruses have also been reported to develop a protection system by developing RNA-silencing viral suppressors (Incarbone and Dunoyer, 2013), even though RNAi does not result in complete gene silencing and requires the components of RNAi to be expressed consistently (Shuey et al., 2002; Rehman et al., 2022). As a result, novel approaches to developing TYLCV-resistant tomato crop varieties are incredibly crucial. CRISPR/Cas technology has proven to be a promising tool for creating designer crop varieties, including pathogen-resistant crops (Shelake et al., 2019; El-Sappah et al., 2021b; Pramanik et al., 2021). The two main components of CRISPR/Cas-based genome editing tools are single guide RNA (sgRNA) and Cas9 endonuclease (Binyameen et al., 2021). The sgRNA-Cas9 complex searches the genome for its target site and uses an adjacent protospacer motif to generate efficient DNA double-strand breaks. During the error-prone DNA repair process, mutations can occur (Pramanik et al., 2021). CRISPR/Cas has recently been used to target either the pathogen genome or the genes of the host plant to achieve a disease-resistant phenotype. By targeting the viral genome, CRISPR/Cas technology is effective in providing TYLCV resistance in *N. benthamiana* (Ali et al., 2015; Zaidi et al., 2016; Tashkandi et al., 2018). Similarly, sgRNAs targeting the *CP* or *Rep* gene decreased TYLCV accumulation in tomato plants (Tashkandi et al., 2018). According to a study published by Pramanik et al. (2021) the commercial tomato BN-86 line was CRISPR/Cas9-interceded to produce TYLCV-resistant tomato plants. Finally, in order to gain viral immunity, targeting the viral genome necessitates the stable expression of the CRISPR/Cas system, and is thus classified as GMO.

## 9 Conclusions

The most destructive viral disease that affects tomatoes is likely TYLCV disease. Traditional strategies, such as reproduction and transgenic techniques, have had limited success in controlling the disease. The QTL for TYLCV resistance, including *Ty-1*, *Ty-2*, *Ty-3*, *Ty-4*, *ty-5*, and *Ty-6* in wild tomato varieties, were only recently discovered. Several methods, including stringent quarantine laws, genetic engineering, conventional breeding, and integrated pest management, have been used to stop the spread of TYLCV. Typically, tomato uses a few defense mechanisms, such as PTI, ETI, Gene silencing, and autophagy, to reduce the dangerous effects of TYLCV infections. This study compiles the characteristics of specific opposition genes, typical opposition resources, subatomic markers for aided choice, and methods for determining TYLCV protection. The main objective is to set the theoretical groundwork for identifying, utilizing, and developing tomato varieties resistant to TYLCV.

## Author contributions

Conceptualization: AE-S, JiaL, KY, MA, AS, MASA, XZ and RM. Draw the figures: AE-S and SQ. Collected the data: AE-S. Contributed to writing the original manuscript draft: AE-S. Review and editing of the manuscript: AE-S, QH, G-TC, JinL, LW, JiaL, MI, XZ, MASA, AS and MA. Writing final copy: AE-S, SS, and ZN. All authors contributed to the article and approved the submitted version.

## References

[B1] Abbas M. Li Y. Elbaiomy R. G. Yan K. Ragauskas A. J. Yadav V. . (2022). Genome-wide analysis and expression profiling of SlHsp70 gene family in solanum lycopersicum revealed higher expression of SlHsp70-11 in roots under Cd(2+) stress. Front. Biosci. (Landmark Ed) 27, 186. doi: 10.31083/j.fbl2706186 35748262

[B2] Abhary M. Anfoka G. Nakhla M. Maxwell D. (2006). Post-transcriptional gene silencing in controlling viruses of the tomato yellow leaf curl virus complex. Arch. Virol. 151, 2349–2363. doi: 10.1007/s00705-006-0819-7 16862387

[B3] Abouzid A. M. Frischmuth T. Jeske H. (1988). A putative replicative form of the abutilon mosaic virus (gemini group) in a chromatin-like structure. Mol. Gen. Genet. MGG 212, 252–258. doi: 10.1007/BF00334693

[B4] Afify A. S. Abdallah M. Ismail S. A. Ataalla M. Abourehab M. A. S. Al-Rashood S. T. . (2022). Development of GC–MS/MS method for environmental monitoring of 49 pesticide residues in food commodities in Al-rass, Al-qassim region, Saudi Arabia. Arabian J. Chem. 15, 104199. doi: 10.1016/j.arabjc.2022.104199

[B5] Al Abdallat A. M. Al Debei H. S. Asmar H. Misbeh S. Quraan A. Kvarnheden A. (2010). An efficient *in vitro*-inoculation method for tomato yellow leaf curl virus. Virol. J. 7, 84. doi: 10.1186/1743-422X-7-84 20429892 PMC2874538

[B6] Albrechtsen S. E. (2006). Testing methods for seed-transmitted viruses: Principles and protocols. UK: CABI Publishing, Oxfordshire), p. 47–81. doi: 10.1079/9780851990163.0001

[B7] Ali Z. Abulfaraj A. Idris A. Ali S. Tashkandi M. Mahfouz M. M. (2015). CRISPR/Cas9-mediated viral interference in plants. Genome Biol. 16, 1–11. doi: 10.1186/s13059-015-0799-6 26556628 PMC4641396

[B8] Alluqmani S. M. Alabdallah N. M. (2022). The effect of thermally heated carbon nanoparticles of oil fly ash on tomato (Solanum lycopersicum l.) under salt stress. J. Soil Sci. Plant Nutr. 22, 5123–5132. doi: 10.07/s42729-022-00988-5

[B9] Amin I. Patil B. L. Briddon R. W. Mansoor S. Fauquet C. M. (2011). Comparison of phenotypes produced in response to transient expression of genes encoded by four distinct begomoviruses in nicotiana benthamiana and their correlation with the levels of developmental miRNAs. Virol. J. 8, 238. doi: 10.1186/1743-422X-8-238 21592402 PMC3166278

[B10] Ammara U. Mansoor S. Saeed M. Amin I. Briddon R. W. Al-Sadi A. M. (2015). RNA Interference-based resistance in transgenic tomato plants against tomato yellow leaf curl virus-Oman (TYLCV-OM) and its associated betasatellite. Virol. J. 12, 38. doi: 10.1186/s12985-015-0263-y 25890080 PMC4359554

[B11] Anbinder I. Reuveni M. Azari R. Paran I. Nahon S. Shlomo H. . (2009). Molecular dissection of tomato leaf curl virus resistance in tomato line TY172 derived from solanum peruvianum. Theor. Appl. Genet. 119, 519–530. doi: 10.1007/s00122-009-1060-z 19455299

[B12] Andersen J. R. Lübberstedt T. (2003). Functional markers in plants. Trends Plant Sci. 8, 554–560. doi: 10.1016/j.tplants.2003.09.010 14607101

[B13] Anfoka G. Moshe A. Fridman L. Amrani L. Rotem O. Kolot M. . (2016). Tomato yellow leaf curl virus infection mitigates the heat stress response of plants grown at high temperatures. Sci. Rep. 6, 1–13. doi: 10.1038/srep19715 26792235 PMC4726131

[B14] Antignus Y. (2007). “The management of tomato yellow leaf curl virus in greenhouses and the open field, a strategy of manipulation,” in Czosnek H (ed.) Tomato yellow leaf curl virus disease. (Dordrecht: Springer) p. 263–278.

[B15] Antignus Y. Vunsh R. Lachman O. Pearlsman M. Maslenin L. Hananya U. . (2004). Truncated rep gene originated from tomato yellow leaf curl virus-Israel [Mild] confers strain-specific resistance in transgenic tomato. Ann. Appl. Biol. 144, 39–44. doi: 10.1111/j.1744-7348.2004.tb00314.x

[B16] Arnholdt-Schmitt B. (2005). Functional markers and a ‘systemic strategy’: convergency between plant breeding, plant nutrition and molecular biology. Plant Physiol. Biochem. 43, 817–820. doi: 10.1016/j.plaphy.2005.08.011 16289946

[B17] Asseri A. H. Alam M. J. Alzahrani F. Khames A. Pathan M. T. Abourehab M. A. S. . (2022). Toward the identification of natural antiviral drug candidates against merkel cell polyomavirus: Computational drug design approaches. Pharmaceuticals 15, 501. doi: 10.3390/ph15050501 35631328 PMC9146542

[B18] AVRDC (2002). “Pyramiding tomato leaf curl virus resistance genes by marker-assisted selection,” in AVRDC report 2001. Eds. Kalb T. Kuo G. (Tainan: Asian Vegetable Research and Development Center), 12–13.

[B19] Bai Y. van der Hulst R. Huang C. Wei L. Stam P. Lindhout P. (2004). Mapping ol-4, a gene conferring resistance to oidium neolycopersici and originating from lycopersicon peruvianum LA2172, requires multi-allelic, single-locus markers. Theor. Appl. Genet. 109, 1215–1223. doi: 10.1007/s00122-004-1698-5 15340683

[B20] Baldodiya G. M. Baruah G. Sen P. Nath P. D. Borah B. K. (2020). “Host-parasite interaction during development of major seed-transmitted viral diseases,” In Kumar R. Gupta A. (eds). Seed-borne diseases of agricultural crops: Detection, diagnosis & management. (Singapore: Springer) p. 265–289. doi: 10.1007/978-981-32-9046-4_11

[B21] Barbieri M. Acciarri N. Sabatini E. Sardo L. Accotto G. Pecchioni N. (2010). Introgression of resistance to two Mediterranean virus species causing tomato yellow leaf curl into a valuable traditional tomato variety. J. Plant Pathol. 92, 485–493.

[B22] Basu S. Kumar Kushwaha N. Kumar Singh A. Pankaj Sahu P. Vinoth Kumar R. Chakraborty S. (2018). Dynamics of a geminivirus-encoded pre-coat protein and host RNA-dependent RNA polymerase 1 in regulating symptom recovery in tobacco. J. Exp. Bot. 69, 2085–2102. doi: 10.1093/jxb/ery043 29432546 PMC6019014

[B23] Bellotti A. C. Arias B. (2001). Host plant resistance to whiteflies with emphasis on cassava as a case study. Crop Prot. 20, 813–823. doi: 10.1016/S0261-2194(01)00113-2

[B24] Ben Tamarzizt H. Gharsallah Chouchane S. Lengliz R. Maxwell D. P. Marrakchi M. Fakhfakh H. . (2009). Use of tomato leaf curl virus (TYLCV) truncated rep gene sequence to engineer TYLCV resistance in tomato plants. Acta Virol. 53, 99–104. doi: 10.4149/av_2009_02_99 19537910

[B25] Bian X.-Y. Thomas M. R. Rasheed M. S. Saeed M. Hanson P. De Barro P. J. . (2007). A recessive allele (tgr-1) conditioning tomato resistance to geminivirus infection is associated with impaired viral movement. Phytopathology 97, 930–937. doi: 10.1094/PHYTO-97-8-0930 18943632

[B26] Binyameen B. Khan Z. Khan S. H. Ahmad A. Munawar N. Mubarik M. S. . (2021). Using multiplexed CRISPR/Cas9 for suppression of cotton leaf curl virus. Int. J. Mol. Sci. 22, 12543. doi: 10.3390/ijms222212543 34830426 PMC8618328

[B27] Bonilla-Ramírez G. Guevara-González R. Garzon-Tiznado J. Ascencio-Ibanez J. Torres-Pacheco I. Rivera-Bustamante R. (1997). Analysis of the infectivity of monomeric clones of pepper huasteco virus. J. Gen. Virol. 78, 947–951. doi: 10.1099/0022-1317-78-4-947 9129670

[B28] Boualem A. Dogimont C. Bendahmane A. (2016). The battle for survival between viruses and their host plants. Curr. Opin. Virol. 17, 32–38. doi: 10.1016/j.coviro.2015.12.001 26800310

[B29] Butterbach P. Verlaan M. G. Dullemans A. Lohuis D. Visser R. G. Bai Y. . (2014). Tomato yellow leaf curl virus resistance by Ty-1 involves increased cytosine methylation of viral genomes and is compromised by cucumber mosaic virus infection. Proc. Natl. Acad. Sci. 111, 12942–12947. doi: 10.1073/pnas.1400894111 25136118 PMC4156758

[B30] Byrne D. N. Bellows T. S. Jr. (1991). Whitefly biology. Annu. Rev. entomol. 36, 431–457. doi: 10.1146/annurev.en.36.010191.002243

[B31] Caro M. Verlaan M. G. Julián O. Finkers R. Wolters A.-M. A. Hutton S. F. . (2015). Assessing the genetic variation of Ty-1 and Ty-3 alleles conferring resistance to tomato yellow leaf curl virus in a broad tomato germplasm. Mol. Breed. 35, 1–13. doi: 10.1007/s11032-015-0329-y PMC4442973 26028987

[B32] Ceniceros-Ojeda E. A. Rodríguez-Negrete E. A. Rivera-Bustamante R. F. (2016). Two populations of viral minichromosomes are present in a geminivirus-infected plant showing symptom remission (recovery). J. Virol. 90, 3828–3838. doi: 10.1128/JVI.02385-15 26792752 PMC4810539

[B33] Chatchawankanphanich O. Maxwell D. P. (2002). Tomato leaf curl karnataka virus from Bangalore, India, appears to be a recombinant begomovirus. Phytopathology 92, 637–645. doi: 10.1094/PHYTO.2002.92.6.637 18944261

[B34] Cheng G. Chang P. Shen Y. Wu L. El-Sappah A. H. Zhang F. . (2020). Comparing the flavor characteristics of 71 tomato (Solanum lycopersicum) accessions in central shaanxi. Front. Plant Sci. 11, 586834. doi: 10.3389/fpls.2020.586834 33362814 PMC7758415

[B35] Chen H. Lin C. Yoshida M. Hanson P. Schafleitner R. (2015). Multiplex PCR for detection of tomato yellow leaf curl disease and root-knot nematode resistance genes in tomato (Solanum lycopersicum l.). Int. J. Plant Breed. Genet. 9, 44–56. doi: 10.3923/ijpbg.2015.44.56

[B36] Chen J. Li W. X. Xie D. Peng J. R. Ding S. W. (2004). Viral virulence protein suppresses RNA silencing–mediated defense but upregulates the role of microRNA in host gene expression. Plant Cell 16, 1302–1313. doi: 10.1105/tpc.018986 15100397 PMC423217

[B37] Cohen S. Harpaz I. (1964). Periodic, rather than continual acquisition of a new tomato virus by its vector, the tobacco whitefly (Bemisia tabaci gennadius) 1. Entomol. exp. Appl. 7, 155–166. doi: 10.1111/j.1570-7458.1964.tb02435.x

[B38] Cohen S. Kern J. Harpaz I. Ben-Joseph R. (1988). Epidemiological studies of the tomato yellow leaf curl virus (TYLCV) in the Jordan valley, Israel. Phytoparasitica 16, 259–270. doi: 10.1007/BF02979527

[B39] Cooper J. Jones A. (1983). Responses of plants to viruses: proposals for the use of terms. Phytopathology 73, 127–128. doi: 10.1094/Phyto-73-127

[B40] Czosnek H. Kheyr-Pour A. Gronenborn B. Remetz E. Zeidan M. Altman A. . (1993). Replication of tomato yellow leaf curl virus (TYLCV) DNA in agroinoculated leaf discs from selected tomato genotypes. Plant Mol. Biol. 22, 995–1005. doi: 10.1007/BF00028972 8400142

[B41] Czosnek H. Laterrot H. (1997). A worldwide survey of tomato yellow leaf curl viruses. Arch. Virol. 142, 1391–1406. doi: 10.1007/s007050050168 9267451

[B42] Dam B. V. Goffau M. D. Lidth De Jeude J. V. Naika S. (2005). Cultivation of tomato: Production, processing and marketing. Available at: https://cgspace.cgiar.org/bitstream/handle/10568/52975/1296_PDF.pdf?sequence=4.

[B43] Davino S. Napoli C. Davino M. Accotto G. P. (2006). Spread of tomato yellow leaf curl virus in Sicily: Partial displacement of another geminivirus originally present. Eur. J. Plant Pathol. 114, 293–299. doi: 10.1007/s10658-005-5805-5

[B44] de Castro A. P. Díez M. J. Nuez F. (2007). Inheritance of tomato yellow leaf curl virus resistance derived from solanum pimpinellifolium UPV16991. Plant Dis. 91, 879–885. doi: 10.1094/PDIS-91-7-0879 30780400

[B45] Delbianco A. Lanzoni C. Klein E. Rubies Autonell C. Gilmer D. Ratti C. (2013). Agroinoculation of b eet necrotic yellow vein virus cDNA clones results in plant systemic infection and efficient p olymyxa betae transmission. Mol. Plant Pathol. 14, 422–428. doi: 10.1111/mpp.12018 23384276 PMC6638874

[B46] de Nazaré Almeida dos Reis L. Fonseca M. E. D. N. Ribeiro S. G. Naito F. Y. B. Boiteux L. S. Pereira-Carvalho R. D. C. (2020). Metagenomics of neotropical single-stranded DNA viruses in tomato cultivars with and without the Ty-1 gene. Viruses 12, 819. doi: 10.3390/v12080819 32731641 PMC7472167

[B47] de Ronde D. Butterbach P. Kormelink R. (2014). Dominant resistance against plant viruses. Front. Plant Sci. 5, 307. doi: 10.3389/fpls.2014.00307 25018765 PMC4073217

[B48] Dhaliwal M. S. Jindal S. K. Sharma A. Prasanna H. (2020). Tomato yellow leaf curl virus disease of tomato and its management through resistance breeding: A review. J. Hortic. Sci. Biotechnol. 95, 425–444. doi: 10.1080/14620316.2019.1691060

[B49] Edris S. Abo-Aba S. Algandaby M. M. Makki R. M. Qari S. H. Al-Quwaie D. A. . (2021). Differential expression of genes contributing to PCD triggered by exogenous oxalic acid in tomato (Solanum lycopersicum). Plant Biosystems-An Int. J. Dealing all Aspects Plant Biol. 155, 871–877. doi: 10.1080/11263504.2020.1810801

[B50] Elebeedy D. Ghanem A. Saleh A. Ibrahim M. H. Kamaly O. A. Abourehab M. A. . (2022). *In vivo* and in silico investigation of the anti-obesity effects of lactiplantibacillus plantarum combined with chia seeds, green tea, and chitosan in alleviating hyperlipidemia and inflammation. Int. J. Mol. Sci. 23, 12200. doi: 10.3390/ijms232012200 36293055 PMC9602495

[B51] Elmore J. M. Lin Z.-J. D. Coaker G. (2011). Plant NB-LRR signaling: upstreams and downstreams. Curr. Opin. Plant Biol. 14, 365–371. doi: 10.1016/j.pbi.2011.03.011 21459033 PMC3155621

[B52] Elmorsy A. E. El-Kassas A. I. Kansouh A. M. Ibraheem M. M. (2021). Selection and breeding new lines of tomato (Solanum lycopersicon l.) resistance to tomato yellow leaf curl virus. Sinai J. Appl. Sci. 10, 99–106. doi: 10.21608/sinjas.2021.75775.1024

[B53] El-Sappah A. H. Elrys A. S. Desoky E.-S. M. Zhao X. Bingwen W. El-Sappah H. H. . (2021a). (eds). “Comprehensive genome wide identification and expression analysis of MTP gene family in tomato (Solanum lycopersicum) under multiple heavy metal stress.” In: Saudi J. Biol. Sci. 2, 6946–6956. doi: 10.1016/j.sjbs.2021.07.073 PMC8626246 34866994

[B54] El-Sappah A. H. Mm I. H El-Awady H. Yan S. Qi S. Liu J. . (2019). Tomato natural resistance genes in controlling the root-knot nematode. Genes 10, 925. doi: 10.3390/genes10110925 31739481 PMC6896013

[B55] El-Sappah A. H. Rather S. A. (2022). “Genomics approaches to study abiotic stress tolerance in plants”. In: Plant Abiotic Stress Physiology, Vol. 2, (Burlington: Apple Academic Press) 2, 25. doi: 10.1201/9781003180579-2

[B56] El-Sappah A. H. Yan K. Huang Q. Islam M. M. Li Q. Wang Y. . (2021b). Comprehensive mechanism of gene silencing and its role in plant growth and development. Front. Plant Sci. 12, 705249. doi: 10.3389/fpls.2021.705249 34589097 PMC8475493

[B57] Friedmann M. Lapidot M. Cohen S. Pilowsky M. (1998). A novel source of resistance to tomato yellow leaf curl virus exhibiting a symptomless reaction to viral infection. J. Am. Soc. Hortic. Sci. 123, 1004–1007. doi: 10.21273/JASHS.123.6.1004

[B58] Fuentes A. Carlos N. Ruiz Y. Callard D. Sánchez Y. Ochagavía M. E. . (2016). Field trial and molecular characterization of RNAi-transgenic tomato plants that exhibit resistance to tomato yellow leaf curl geminivirus. Mol. Plant-Microbe Interact. 29, 197–209. doi: 10.1094/MPMI-08-15-0181-R 26713353

[B59] Garcia B. E. Graham E. Jensen K. S. Hanson P. Mejía L. Maxwell D. P. (2007). Co-Dominant SCAR marker for detection of the begomovirus-resistance Ty-2 locus derived from solanum habrochaites in tomato germplasm. Tomato Genet. Coop. Rep. 57, 21–24.

[B60] Garzón-Tiznado J. A. Torres-Pacheco I. Ascencio-Iba Ez J. Herrera-Estrella L. (1993). Inoculation of peppers with infectious clones of a new geminivirus by a biolistic procedure. Phytopathology-New York And Baltimore Then St Paul- 83, 514–514. doi: 10.1094/Phyto-83-514

[B61] Gill U. Scott J. W. Shekasteband R. Ogundiwin E. Schuit C. Francis D. M. . (2019). Ty-6, a major begomovirus resistance gene on chromosome 10, is effective against tomato yellow leaf curl virus and tomato mottle virus. Theor. Appl. Genet. 132, 1543–1554. doi: 10.1007/s00122-019-03298-0 30758531 PMC6476845

[B62] Gnanasekaran P. Kishorekumar R. Bhattacharyya D. Vinoth Kumar R. Chakraborty S. (2019). Multifaceted role of geminivirus associated betasatellite in pathogenesis. Mol. Plant Pathol. 20, 1019–1033. doi: 10.1111/mpp.12800 31210029 PMC6589721

[B63] Gorovits R. Moshe A. Ghanim M. Czosnek H. (2013). Recruitment of the host plant heat shock protein 70 by tomato yellow leaf curl virus coat protein is required for virus infection. PloS One 8, e70280. doi: 10.1371/journal.pone.0070280 23894631 PMC3720902

[B64] Grimsley N. Hohn B. Hohn T. Walden R. (1986). “Agroinfection,” an alternative route for viral infection of plants by using the Ti plasmid. Proc. Natl. Acad. Sci. 83, 3282–3286. doi: 10.1073/pnas.83.10.3282 16593697 PMC323497

[B65] Gronenborn B. (2007). “The tomato yellow leaf curl virus genome and function of its proteins,” IIn Czosnek H. (eds.) Tomato yellow leaf curl virus disease. (Dordrecht: Springer), 67–84. doi: 10.1007/978-1-4020-4769-5_5

[B66] Guo J. Sun K. Zhang Y. Hu K. Zhao X. Liu H. . (2021). SlMAPK3, a key mitogen-activated protein kinase, regulates the resistance of cherry tomato fruit to botrytis cinerea induced by yeast cell wall and β-glucan. Postharvest Biol. Technol. 171, 111350. doi: 10.1016/j.postharvbio.2020.111350

[B67] Gupta N. Reddy K. Bhattacharyya D. (2021). Plant responses to geminivirus infection: guardians of the plant immunity. Virol. J. 18, 1–25. doi: 10.1186/s12985-021-01612-1 34243802 PMC8268416

[B68] Gutierrez C. (1999). Geminivirus DNA replication. Cell. Mol. Life Sci. CMLS 56, 313–329. doi: 10.1007/s000180050433 11212359 PMC11146802

[B69] Hafrén A. Macia J.-L. Love A. J. Milner J. J. Drucker M. Hofius D. (2017). Selective autophagy limits cauliflower mosaic virus infection by NBR1-mediated targeting of viral capsid protein and particles. Proc. Natl. Acad. Sci. 114, E2026–E2035. doi: 10.1073/pnas.1610687114 28223514 PMC5347569

[B70] Hanley-Bowdoin L. Bejarano E. R. Robertson D. Mansoor S. (2013). Geminiviruses: masters at redirecting and reprogramming plant processes. Nat. Rev. Microbiol. 11, 777–788. doi: 10.1038/nrmicro3117 24100361

[B71] Hanley-Bowdoin L. Settlage S. B. Robertson D. (2004). Reprogramming plant gene expression: a prerequisite to geminivirus DNA replication. Mol. Plant Pathol. 5, 149–156. doi: 10.1111/j.1364-3703.2004.00214.x 20565592

[B72] Hanson P. M. Bernacchi D. Green S. Tanksley S. D. Muniyappa V. Padmaja A. S. . (2000). Mapping a wild tomato introgression associated with tomato yellow leaf curl virus resistance in a cultivated tomato line. J. Am. Soc. Hortic. Sci. 125, 15–20. doi: 10.21273/JASHS.125.1.15

[B73] Hanson P. Green S. Kuo G. (2006). Ty-2, a gene on chromosome 11 conditioning geminivirus resistance in tomato. Tomato Genet. Coop Rep. 56, 17–18.

[B74] Hanson P. Lu S.-F. Wang J.-F. Chen W. Kenyon L. Tan C.-W. . (2016). Conventional and molecular marker-assisted selection and pyramiding of genes for multiple disease resistance in tomato. Scientia Hortic. 201, 346–354. doi: 10.1016/j.scienta.2016.02.020

[B75] Haxim Y. Ismayil A. Jia Q. Wang Y. Zheng X. Chen T. . (2017). Autophagy functions as an antiviral mechanism against geminiviruses in plants. Elife 6, e23897. doi: 10.7554/eLife.23897.024 28244873 PMC5362266

[B76] Howladar S. M. (2016). Exogenous applications of biochar and a-tocopherol improve the performance of salt-stressed tomato plants. Umm Al-Qura Univ. J. Appl. Sci. (UQUJAS) 3 (1), 16.

[B77] Hussain I. Farooq T. Khan S. Ali N. Waris M. Jalal A. . (2022). Variability in indigenous Pakistani tomato lines and worldwide reference collection for tomato mosaic virus (ToMV) and tomato yellow leaf curl virus (TYLCV) infection. Braz. J. Biol. 84, 2024. doi: 10.1590/1519-6984.253605 35137839

[B78] Hutton S. F. Scott J. W. (2013). Fine-mapping and cloning of Ty-1 and Ty-3; and mapping of a new TYLCV resistance locus,”Ty-6”. In: Tomato Breeders Round Table Proceedings 2013, Chiang Mai. Available at: https://tgc.ifas.ufl.edu/2013/abstracts/SamOrchardAbstract%20TBRT%202013.pdf

[B79] Hutton S. Scott J. (2014). Ty-6, a major begomovirus resistance gene located on chromosome 10. Rept. Tomato Genet. Coop 64, 14–18. 10.1007/s00122-019-03298-0 PMC6476845 30758531

[B80] Hutton S. F. Scott J. W. Schuster D. J. (2012). Recessive resistance to tomato yellow leaf curl virus from the tomato cultivar tyking is located in the same region as Ty-5 on chromosome 4. HortScience 47, 324–327. doi: 10.21273/HORTSCI.47.3.324

[B81] Hu X. Wang R. Li F. Zhou T. Yang W. (2014). Development of new marker for tomato TYLCV resistant gene and its application in selection of multi resistant gene pyramiding. China Veg. 10, 18–23.

[B82] Ibne-Siam-Joy M. (2020). Assessment of varietal performance of selected tomato varieties against tomato yellow leaf curl virus (Tylcv) and its molecular detection through pcr. Department Of Plant Pathol. 13-05417, 1–73.

[B83] Idris A. Brown J. (2005). Evidence for interspecific-recombination for three monopartite begomoviral genomes associated with the tomato leaf curl disease from central Sudan. Arch. Virol. 150, 1003–1012. doi: 10.1007/s00705-004-0484-7 15703848

[B84] Incarbone M. Dunoyer P. (2013). RNA Silencing and its suppression: novel insights from in planta analyses. Trends Plant Sci. 18, 382–392. doi: 10.1016/j.tplants.2013.04.001 23684690

[B85] Ioannou N. (1985). Yellow leaf curl and other virus diseases of tomato in Cyprus. Plant Pathol. 34, 428–434. doi: 10.1111/j.1365-3059.1985.tb01383.x

[B86] Islam M. Qi S. Zhang S. Amin B. Yadav V. El-Sappah A. H. . (2022). Genome-wide identification and functions against tomato spotted wilt tospovirus of PR-10 in solanum lycopersicum. Int. J. Mol. Sci. 23, 1502. doi: 10.3390/ijms23031502 35163430 PMC8835967

[B87] Jansson G. Hansen J. K. Haapanen M. Kvaalen H. Steffenrem A. (2017). The genetic and economic gains from forest tree breeding programmes in Scandinavia and Finland. Scand. J. For. Res. 32, 273–286. doi: 10.1080/02827581.2016.1242770

[B88] Jensen K. Van Betteray B. Smeets J. Yuanfu J. Scott J. Mejia L. . (2007). Co Dominant SCAR marker, P6-25, for detection of the ty-3, Ty-3, and Ty-3a alleles at 25 cM of chromosome 6 of tomato. College of Agricultural and Life Sciences at University of Wisconsin-Madison, and by grants from Unilever Bestfoods Ltd. and the Florida Tomato Committee to JW. Scott. p.25

[B89] Jia Y. Lisch D. R. Ohtsu K. Scanlon M. J. Nettleton D. Schnable P. S. (2009). Loss of RNA–dependent RNA polymerase 2 (RDR2) function causes widespread and unexpected changes in the expression of transposons, genes, and 24-nt small RNAs. PloS Genet. 5, e1000737. doi: 10.1371/journal.pgen.1000737 19936292 PMC2774947

[B90] Jiang G.-L. (2013). Molecular markers and marker-assisted breeding in plants. In: Andersen S. B (ed). Plant Breeding from Laboratories to Fields (Intech) p. 45–83. doi: 10.5772/52583

[B91] Ji Y. Schuster D. J. Scott J. W. (2007a). Ty-3, a begomovirus resistance locus near the tomato yellow leaf curl virus resistance locus Ty-1 on chromosome 6 of tomato. Mol. Breed. 20, 271–284. doi: 10.1007/s11032-007-9089-7

[B92] Ji Y. Scott J. W. Hanson P. Graham E. Maxwell D. P. (2007b). “Sources of resistance, inheritance, and location of genetic loci conferring resistance to members of the tomato-infecting begomoviruses,” in Tomato Yellow Leaf Curl Virus Disease: Management, Molecular Biology, Breeding for Resistance Czosnek H. (ed.) (Dordrecht: Springer) p. 343–362. doi: 10.1007/978-1-4020-4769-5_20

[B93] Ji Y. Scott J. W. Schuster D. J. (2009a). Toward fine mapping of the tomato yellow leaf curl virus resistance gene Ty-2 on chromosome 11 of tomato. HortScience 44, 614–618. doi: 10.21273/HORTSCI.44.3.614 PMC4092234 25076841

[B94] Ji Y. Scott J. W. Schuster D. J. Maxwell D. P. (2009b). Molecular mapping of Ty-4, a new tomato yellow leaf curl virus resistance locus on chromosome 3 of tomato. J. Am. Soc. Hortic. Sci. 134, 281–288. doi: 10.21273/JASHS.134.2.281

[B95] Jones J. D. Dangl J. L. (2006). The plant immune system. Nature 444, 323–329. doi: 10.1038/nature05286 17108957

[B96] Jung J. Kim H. J. Lee J. M. Oh C. S. Lee H.-J. Yeam I. (2015). Gene-based molecular marker system for multiple disease resistances in tomato against tomato yellow leaf curl virus, late blight, and verticillium wilt. Euphytica 205, 599–613. doi: 10.1007/s10681-015-1442-z

[B97] Kadirvel P. de la Peña R. Schafleitner R. Huang S. Geethanjali S. Kenyon L. . (2013). Mapping of QTLs in tomato line FLA456 associated with resistance to a virus causing tomato yellow leaf curl disease. Euphytica 190, 297–308. doi: 10.1007/s10681-012-0848-0

[B98] Kalloo G. Banerjee M. (1990). Transfer of tomato leaf curl virus resistance from Lycopersicon hirsutum. Plant Breed. 105, 156–159. doi: 10.1111/j.1439-0523.1990.tb00469.x

[B99] Kasai K. Morikawa Y. Sorri V. Valkonen J. Gebhardt C. Watanabe K. (2000). Development of SCAR markers to the PVY resistance gene ry adg based on a common feature of plant disease resistance genes. Genome 43, 1–8. doi: 10.1139/g99-092 10701106

[B100] Kegler H. (1994). Incidence, properties and control of tomato yellow leaf curl virus-a review. Arch. Phytopathol. Plant Prot. 29, 119–132. doi: 10.1080/03235409409383102

[B101] Kheyr-Pour A. Gronenborn B. Czosner H. (1994). Agroinoculation of tomato yellow leaf curl virus (TYLCV) overcomes the virus resistance of wild lycopersicon species. Plant Breed. 112, 228–233. doi: 10.1111/j.1439-0523.1994.tb00675.x

[B102] Kil E.-J. Kim S. Lee Y.-J. Byun H.-S. Park J. Seo H. . (2016). Tomato yellow leaf curl virus (TYLCV-IL): A seed-transmissible geminivirus in tomatoes. Sci. Rep. 6, 1–10. doi: 10.1038/srep19013 26743765 PMC4705557

[B103] Kil E.-J. Park J. Choi E.-Y. Byun H.-S. Lee K.-Y. An C. G. . (2018). Seed transmission of tomato yellow leaf curl virus in sweet pepper (Capsicum annuum). Eur. J. Plant Pathol. 150, 759–764. doi: 10.1007/s10658-017-1304-8

[B104] Kil E.-J. Park J. Choi H.-S. Kim C.-S. Lee S. (2017). Seed transmission of tomato yellow leaf curl virus in white soybean (Glycine max). Plant Pathol. J. 33, 424. doi: 10.5423/PPJ.NT.02.2017.0043 28811759 PMC5538446

[B105] Kim M. Park Y. Lee J. Sim S.-C. (2020). Development of molecular markers for Ty-2 and Ty-3 selection in tomato breeding. Scientia Hortic. 265, 109230. doi: 10.1016/j.scienta.2020.109230

[B106] Koeda S. Fujiwara I. Oka Y. Kesumawati E. Zakaria S. Kanzaki S. (2020). Ty-2 and Ty-3a conferred resistance are insufficient against tomato yellow leaf curl kanchanaburi virus from southeast Asia in single or mixed infections of tomato. Plant Dis. 104, 3221–3229. doi: 10.1094/PDIS-03-20-0613-RE 33044916

[B107] Lapidot M. (2007). “Screening for TYLCV-resistance plants using whitefly-mediated inoculation,” In: Czosnek H. (eds.) Tomato yellow leaf curl virus disease (Dordrecht: Springer). doi: 10.1007/978-1-4020-4769-5_19.

[B108] Lapidot M. Friedmann M. Lachman O. Yehezkel A. Nahon S. Cohen S. . (1997). Comparison of resistance level to tomato yellow leaf curl virus among commercial cultivars and breeding lines. Plant Dis. 81, 1425–1428. doi: 10.1094/PDIS.1997.81.12.1425 30861796

[B109] Lapidot M. Friedmann M. Pilowsky M. Ben-Joseph R. Cohen S. (2001). Effect of host plant resistance to tomato yellow leaf curl virus (TYLCV) on virus acquisition and transmission by its whitefly vector. Phytopathology 91, 1209–1213. doi: 10.1094/PHYTO.2001.91.12.1209 18943336

[B110] Lapidot M. Karniel U. Gelbart D. Fogel D. Evenor D. Kutsher Y. . (2015). A novel route controlling begomovirus resistance by the messenger RNA surveillance factor pelota. PloS Genet. 11, e1005538. doi: 10.1371/journal.pgen.1005538 26448569 PMC4598160

[B111] Lapidot M. Polston J. E. (2006). “Resistance to tomato yellow leaf curl virus in tomato,” In: Loebenstein G. Carr J.P. (eds.) Natural resistance mechanisms of plants to viruses (Dordrecht: Springer), 503–520. doi: 10.1007/1-4020-3780-5_23

[B112] Laterrot H. (1993). “Present state of the genetic control of tomato yellow leaf curl virus and of the EEC-supported breeding programme.” In: Proceedings of the Xiith Eucarpia Meeting on Tomato Genetics and Breeding (ed. Stamova L ) pp. 27–31. Plovdiv (BG)

[B113] Lee J. H. Chung D. J. Lee J. M. Yeam I. (2020). Development and application of gene-specific markers for tomato yellow leaf curl virus resistance in both field and artificial infections. Plants 10, 9. doi: 10.3390/plants10010009 33374801 PMC7824369

[B114] Legarrea S. Barman A. Diffie S. Srinivasan R. (2020). Virus accumulation and whitefly performance modulate the role of alternate host species as inoculum sources of tomato yellow leaf curl virus. Plant Dis. 104, 2958–2966. doi: 10.1094/PDIS-09-19-1853-RE 32897844

[B115] Leibman D. Prakash S. Wolf D. Zelcer A. Anfoka G. Haviv S. . (2015). Immunity to tomato yellow leaf curl virus in transgenic tomato is associated with accumulation of transgene small RNA. Arch. Virol. 160, 2727–2739. doi: 10.1007/s00705-015-2551-7 26255053

[B116] León J. Lawton M. A. Raskin I. (1995). Hydrogen peroxide stimulates salicylic acid biosynthesis in tobacco. Plant Physiol. 108, 1673–1678. doi: 10.1104/pp.108.4.1673 12228572 PMC157549

[B117] Levin I. Karniel U. Fogel D. Reuveni M. Gelbart D. Evenor D. . (2013). “Cloning and analysis of the tomato yellow leaf curl virus resistance gene Ty-5,” in Proceedings of the tomato breeders roundtable, Chaing-Mai, Thailand. Available at: http://tgc.ifas.ufl.edu/2013/abstracts/LevinAbstract%20TBRT%202013.pdf.

[B118] Li Y. Qin L. Zhao J. Muhammad T. Cao H. Li H. . (2017). SlMAPK3 enhances tolerance to tomato yellow leaf curl virus (TYLCV) by regulating salicylic acid and jasmonic acid signaling in tomato (Solanum lycopersicum). PloS One 12, e0172466. doi: 10.1371/journal.pone.0172466 28222174 PMC5319765

[B119] Liu M. A. (2011). DNA Vaccines: An historical perspective and view to the future. Immunol. Rev. 239, 62–84. doi: 10.1111/j.1600-065X.2010.00980.x 21198665

[B120] Liu J. Shi M. Wang J. Zhang B. Li Y. Wang J. . (2020). Comparative transcriptomic analysis of the development of sepal morphology in tomato (Solanum lycopersicum l.). Int. J. Mol. Sci. 21, 5914. doi: 10.3390/ijms21165914 32824631 PMC7460612

[B121] Loriato V. A. P. Martins L. G. C. Euclydes N. C. Reis P. A. B. Duarte C. E. M. Fontes E. P. B. (2020). Engineering resistance against geminiviruses: A review of suppressed natural defenses and the use of RNAi and the CRISPR/Cas system. Plant Sci. 292, 110410. doi: 10.1016/j.plantsci.2020.110410 32005374

[B122] Lucioli A. Noris E. Brunetti A. Tavazza R. Ruzza V. Castillo A. G. . (2003). Tomato yellow leaf curl Sardinia virus rep-derived resistance to homologous and heterologous geminiviruses occurs by different mechanisms and is overcome if virus-mediated transgene silencing is activated. J. Virol. 77, 6785–6798. doi: 10.1128/JVI.77.12.6785-6798.2003 12767999 PMC156158

[B123] Lu R. Martin-Hernandez A. M. Peart J. R. Malcuit I. Baulcombe D. C. (2003). Virus-induced gene silencing in plants. Methods 30, 296–303. doi: 10.1016/S1046-2023(03)00037-9 12828943

[B124] Luna A. P. Rodríguez-Negrete E. A. Morilla G. Wang L. Lozano-Durán R. Castillo A. G. . (2017). V2 from a curtovirus is a suppressor of post-transcriptional gene silencing. J. Gen. Virol. 98, 2607–2614. doi: 10.1099/jgv.0.000933 28933688

[B125] Luna A. P. Romero-Rodríguez B. Rosas-Díaz T. Cerero L. Rodríguez-Negrete E. A. Castillo A. G. . (2020). Characterization of curtovirus V2 protein, a functional homolog of begomovirus V2. Front. Plant Sci. 11, 835. doi: 10.3389/fpls.2020.00835 32636860 PMC7318802

[B126] Macho A. P. Zipfel C. (2015). Targeting of plant pattern recognition receptor-triggered immunity by bacterial type-III secretion system effectors. Curr. Opin. Microbiol. 23, 14–22. doi: 10.1016/j.mib.2014.10.009 25461568

[B127] Makkouk K. Shehab S. Majdalani S. (1979). Tomato yellow leaf curl: incidence, yield and losses and transmission in Lebanon. Phytopathol. Z. 96, 263–267. doi: 10.1111/j.1439-0434.1979.tb01648.x

[B128] Maliano M. R. (2021). Investigation of the resistance and susceptible responses of tomato (Solanum lycopersicum) to tomato yellow leaf curl virus, the invasion biology of tomato begomoviruses in Costa Rica and characterization of two weed-infecting begomoviruses in the Caribbean basin (Davis: University of California).

[B129] Mauck K. E. (2016). Variation in virus effects on host plant phenotypes and insect vector behavior: what can it teach us about virus evolution? Curr. Opin. Virol. 21, 114–123. doi: 10.1016/j.coviro.2016.09.002 27644035

[B130] Ma X. Xu G. He P. Shan L. (2016). SERKing coreceptors for receptors. Trends Plant Sci. 21, 1017–1033. doi: 10.1016/j.tplants.2016.08.014 27660030

[B131] Mazier M. German-Retana S. Flamain F. Dubois V. Botton E. Sarnette V. . (2004). A simple and efficient method for testing lettuce mosaic virus resistance in *in vitro* cultivated lettuce. J. Virol. Methods 116, 123–131. doi: 10.1016/j.jviromet.2003.11.011 14738978

[B132] Migicovsky Z. Myles S. (2017). Exploiting wild relatives for genomics-assisted breeding of perennial crops. Front. Plant Sci. 8, 460. doi: 10.3389/fpls.2017.00460 28421095 PMC5379136

[B133] Milo J. (2001). “The PCR-based marker REX-1, linked to the gene mi, can be used as a marker to TYLCV tolerance,” in Proc Tomato Breed Roundtable, Antigua. Available at: http://www.oardc.ohiostate.edu/tomato/TBRT%202001%20Abstracts.pdf.

[B134] Mori N. Hasegawa S. Takimoto R. Horiuchi R. Watanabe C. Onizaki D. . (2022). Identification of QTLs conferring resistance to begomovirus isolate of PepYLCIV in capsicum chinense. Euphytica 218, 1–12. doi: 10.1007/s10681-022-02970-9

[B135] Morilla G. Janssen D. García-Andrés S. Moriones E. Cuadrado I. Bejarano E. (2005). Pepper (Capsicum annuum) is a dead-end host for tomato yellow leaf curl virus. Phytopathology 95, 1089–1097. doi: 10.1094/PHYTO-95-1089 18943307

[B136] Morinaka Y. Sakamoto T. Inukai Y. Agetsuma M. Kitano H. Ashikari M. . (2006). Morphological alteration caused by brassinosteroid insensitivity increases the biomass and grain production of rice. Plant Physiol. 141, 924–931. doi: 10.1104/pp.106.077081 16714407 PMC1489896

[B137] Mori T. Takenaka K. Domoto F. Aoyama Y. Sera T. (2021). Development of a method to rapidly assess resistance/susceptibility of micro-tom tomatoes to tomato yellow leaf curl virus *via* agroinoculation of cotyledons. BMC Res. Notes 14, 237. doi: 10.1186/s13104-021-05651-3 34162412 PMC8220776

[B138] Morris M. Dreher K. Ribaut J.-M. Khairallah M. (2003). Money matters (II): costs of maize inbred line conversion schemes at CIMMYT using conventional and marker-assisted selection. Mol. Breed. 11, 235–247. doi: 10.1023/A:1022872604743

[B139] Mourrain P. Béclin C. Elmayan T. Feuerbach F. Godon C. Morel J.-B. . (2000). Arabidopsis SGS2 and SGS3 genes are required for posttranscriptional gene silencing and natural virus resistance. Cell 101, 533–542. doi: 10.1016/S0092-8674(00)80863-6 10850495

[B140] Nakhla M. Sorensen A. Mejía L. Ramírez P. Karkashian J. Maxwell D. (2005). Molecular characterization of tomato-infecting begomoviruses in Central America and development of DNA-based detection methods. Acta Hortic 695, 277–288. doi: 10.17660/ActaHortic.2005.695.31

[B141] Navas-Castillo J. Sánchez-Campos S. Díaz J. Sáez-Alonso E. Moriones E. (1997). First report of tomato yellow leaf curl virus-is in Spain: coexistence of two different geminiviruses in the same epidemic outbreak. Plant Dis. 81, 1461–1461. doi: 10.1094/PDIS.1997.81.12.1461B 30861805

[B142] Nehra C. Verma R. K. Petrov N. M. Stoyanova M. I. Sharma P. Gaur R. K. (2022). Computational analysis for plant virus analysis using next-generation sequencing. Bioinf. Agric. 14, 383–398. doi: 10.1016/B978-0-323-89778-5.00013-1

[B143] Nevame A. Y. M. Xia L. Nchongboh C. G. Hasan M. M. Alam M. Yongbo L. . (2018). Development of a new molecular marker for the resistance to tomato yellow leaf curl virus. BioMed. Res. Int. 2018, 812028. doi: 10.1155/2018/8120281 PMC6076955 30105248

[B144] Ning W. Shi X. Liu B. Pan H. Wei W. Zeng Y. . (2015). Transmission of tomato yellow leaf curl virus by bemisia tabaci as affected by whitefly sex and biotype. Sci. Rep. 5, 1–8. doi: 10.1038/srep10744 PMC4448267 26021483

[B145] Pakkianathan B. C. Kontsedalov S. Lebedev G. Mahadav A. Zeidan M. Czosnek H. . (2015). Replication of tomato yellow leaf curl virus in its whitefly vector, bemisia tabaci. J. Virol. 89, 9791–9803. doi: 10.1128/JVI.00779-15 26178995 PMC4577905

[B146] Pan H. Chu D. Yan W. Su Q. Liu B. Wang S. . (2012). Rapid spread of tomato yellow leaf curl virus in China is aided differentially by two invasive whiteflies. PloS One 7, e34817. doi: 10.1371/journal.pone.0034817 22514670 PMC3325912

[B147] Peleg-Grossman S. Melamed-Book N. Cohen G. Levine A. (2010). Cytoplasmic H2O2 prevents translocation of NPR1 to the nucleus and inhibits the induction of PR genes in arabidopsis. Plant Signaling Behav. 5, 1401–1406. doi: 10.4161/psb.5.11.13209 PMC3115241 21051935

[B148] Péréfarres F. Lefeuvre P. Hoareau M. Thierry M. Magali D. Reynaud B. . (2010). Rapid displacement as a result of interaction between strains of TYLCV in reunion island. Acta Hortic. 914, 197–201. doi: 10.17660/ActaHortic.2011.914.36

[B149] Pereira-Carvalho R. C. Díaz-Pendón J. A. Fonseca M. E. N. Boiteux L. S. Fernández-Muñoz R. Moriones E. . (2015). Recessive resistance derived from tomato cv. tyking-limits drastically the spread of tomato yellow leaf curl virus. Viruses 7, 2518–2533. doi: 10.3390/v7052518 26008699 PMC4452918

[B150] Pérez de Castro A. Julián O. Díez M. J. (2013). Genetic control and mapping of solanum chilense LA1932, LA1960 and LA1971-derived resistance to tomato yellow leaf curl disease. Euphytica 190, 203–214. doi: 10.1007/s10681-012-0792-z

[B151] Picó B. Díez M. Nuez F. (1998). Evaluation of whitefly-mediated inoculation techniques to screen lycopersicon esculentum and wild relatives for resistance to tomato yellow leaf curl virus. Euphytica 101, 259–271. doi: 10.1023/A:1018353806051

[B152] Pico B. Ferriol M. Diez M. Vinals F. (2001). Agroinoculation methods to screen wild lycopersicon for resistance to tomato yellow leaf curl virus. J. Plant Pathol. 83, 215–220. doi: 10.2307/41998064

[B153] Picó B. Herraiz J. Ruiz J. Nuez F. (2002). Widening the genetic basis of virus resistance in tomato. Scientia Hortic. 94, 73–89. doi: 10.1016/S0304-4238(01)00376-4

[B154] Picó B. Sifres A. Elía M. Díez M. J. Nuez F. (2000). Searching for new resistance sources to tomato yellow leaf curl virus within a highly variable wild lycopersicon genetic pool. Acta Physiol. Plantarum 22, 344–350. doi: 10.1007/s11738-000-0051-0

[B155] Piedra-Aguilera Á. Jiao C. Luna A. P. Villanueva F. Dabad M. Esteve-Codina A. . (2019). Integrated single-base resolution maps of transcriptome, sRNAome and methylome of tomato yellow leaf curl virus (TYLCV) in tomato. Sci. Rep. 9, 1–16. doi: 10.1038/s41598-019-39239-6 30814535 PMC6393547

[B156] Poczai P. Varga I. Laos M. Cseh A. Bell N. Valkonen J. . (2013). Advances in plant gene-targeted and functional markers: A review. Plant Methods 9, 1–32. doi: 10.1186/1746-4811-9-6 23406322 PMC3583794

[B157] Polston J. E. Lapidot M. (2007). “Management of tomato yellow leaf curl virus: US and Israel perspectives,” In: Czosnek H. (Eds) Tomato yellow leaf curl virus disease (Dordrecht: Springer), 251–262. doi: 10.1007/978-1-4020-4769-5_15

[B158] Pozharskiy A. Kostyukova V. Taskuzhina A. Nizamdinova G. Kisselyova N. Kalendar R. . (2022). Screening a collection of local and foreign varieties of solanum lycopersicum l. in Kazakhstan for genetic markers of resistance against three tomato viruses. Heliyon 8, e10095. doi: 10.1016/j.heliyon.2022.e10095 36033267 PMC9399970

[B159] Prabhandakavi P. Pogiri R. Kumar R. Acharya S. Esakky R. Chakraborty M. . (2021). Pyramiding Ty-1/Ty-3, Ty-2, ty-5 and ty-6 genes into tomato hybrid to develop resistance against tomato leaf curl viruses and recurrent parent genome recovery by ddRAD sequencing method. J. Plant Biochem. Biotechnol. 30, 462–476. doi: 10.1007/s13562-020-00633-1

[B160] Pramanik D. Shelake R. M. Park J. Kim M. J. Hwang I. Park Y. . (2021). CRISPR/Cas9-mediated generation of pathogen-resistant tomato against tomato yellow leaf curl virus and powdery mildew. Int. J. Mol. Sci. 22, 1878. doi: 10.3390/ijms22041878 33668636 PMC7917697

[B161] Prasad A. Sharma N. Hari-Gowthem G. Muthamilarasan M. Prasad M. (2020). Tomato yellow leaf curl virus: impact, challenges, and management. Trends Plant Sci. 25, 897–911. doi: 10.1016/j.tplants.2020.03.015 32371058

[B162] Prasanna H. Sinha D. Rai G. Krishna R. Kashyap S. Singh N. . (2015). Pyramiding T y-2 and T y-3 genes for resistance to monopartite and bipartite tomato leaf curl viruses of I ndia. Plant Pathol. 64, 256–264. doi: 10.1111/ppa.12267

[B163] Qi X. Bao F. S. Xie Z. (2009). Small RNA deep sequencing reveals role for arabidopsis thaliana RNA-dependent RNA polymerases in viral siRNA biogenesis. PloS One 4, e4971. doi: 10.1371/annotation/8d1a816e-b366-4833-b558-724ec28d1b87 19308254 PMC2654919

[B164] Qi S. Zhang S. Islam M. M. El-Sappah A. H. Zhang F. Liang Y. (2021). Natural resources resistance to tomato spotted wilt virus (TSWV) in tomato (Solanum lycopersicum). Int. J. Mol. Sci. 22 (20), 10978. doi: 10.3390/ijms222010978 34681638 PMC8538096

[B165] Quamruzzaman A. Islam F. Mallick S. R. (2021). Insect and diseases resistance in tomato entries. Am. J. Plant Sci. 12, 1646–1657. doi: 10.4236/ajps.2021.1211115

[B166] Raja P. Wolf J. N. Bisaro D. M. (2010). RNA Silencing directed against geminiviruses: post-transcriptional and epigenetic components. Biochim. Biophys. Acta (BBA)-Gene Regul. Mech. 1799, 337–351. doi: 10.1016/j.bbagrm.2010.01.004 20079472

[B167] Ramkumar G. Sivaranjani A. Pandey M. K. Sakthivel K. Shobha Rani N. Sudarshan I. . (2010). Development of a PCR-based SNP marker system for effective selection of kernel length and kernel elongation in rice. Mol. Breed. 26, 735–740. doi: 10.1007/s11032-010-9492-3

[B168] Ramkumar G. Srinivasarao K. Mohan K. M. Sudarshan I. Sivaranjani A. Gopalakrishna K. . (2011). Development and validation of functional marker targeting an InDel in the major rice blast disease resistance gene Pi54 (Pik h). Mol. Breed. 27, 129–135. doi: 10.1007/s11032-010-9538-6

[B169] Ramos P. Guevara-Gonzalez R. Peral R. Ascencio-Ibanez J. Polston J. Argüello-Astorga G. . (2003). Tomato mottle taino virus pseudorecombines with PYMV but not with ToMoV: implications for the delimitation of cis-and trans-acting replication specificity determinants. Arch. Virol. 148, 1697–1712. doi: 10.1007/s00705-003-0136-3 14505083

[B170] Rasheed A. Raza A. Jie H. Mahmood A. Ma Y. Zhao L. . (2022). Molecular tools and their applications in developing salt-tolerant soybean (Glycine max l.) cultivars. Bioengineering 9, 495. doi: 10.3390/bioengineering9100495 36290463 PMC9598088

[B171] Raza A. Salehi H. Rahman M. A. Zahid Z. Madadkar Haghjou M. Najafi-Kakavand S. . (2022). Plant hormones and neurotransmitter interactions mediate antioxidant defenses under induced oxidative stress in plants. Front. Plant Sci. 13. doi: 10.3389/fpls.2022.961872 PMC9514553 36176673

[B172] Rehman U. Parveen N. Sheikh A. Abourehab M. A. S. Sahebkar A. Kesharwani P. (2022). Polymeric nanoparticles-siRNA as an emerging nano-polyplexes against ovarian cancer. Colloids Surfaces B: Biointerfaces 218, 112766. doi: 10.1016/j.colsurfb.2022.112766 35994990

[B173] Ren Y. Tao X. Li D. Yang X. Zhou X. (2022). Ty-5 confers broad-spectrum resistance to geminiviruses. Viruses 14, 1804. doi: 10.3390/v14081804 36016426 PMC9415776

[B174] Reyes M. I. Nash T. E. Dallas M. M. Ascencio-Ibáñez J. T. Hanley-Bowdoin L. (2013). Peptide aptamers that bind to geminivirus replication proteins confer a resistance phenotype to tomato yellow leaf curl virus and tomato mottle virus infection in tomato. J. Virol. 87, 9691–9706. doi: 10.1128/JVI.01095-13 23824791 PMC3754110

[B175] Roselló S. Nuez F. (1999). Estado actual de la lucha contra el virus del bronceado en el tomate. Vida Rural 90, 48–52.

[B176] Roy A. (2016). Abstracts of the 8th international geminivirus symposium and the 6th international ssDNA comparative virology workshop, 7–10th November 2016, new Delhi. VirusDisease 27, 405–459. doi: 10.1007/s13337-016-0351-7

[B177] Russo P. Slack S. (1998). Tissue culture methods for the screening and analysis of putative virus-resistant transgenic potato plants. Phytopathology 88, 437–441. doi: 10.1094/PHYTO.1998.88.5.437 18944923

[B178] Saeed M. Briddon R. W. Dalakouras A. Krczal G. Wassenegger M. (2015). Functional analysis of cotton leaf curl kokhran Virus/Cotton leaf curl multan betasatellite RNA silencing suppressors. Biol. (Basel) 4, 697–714. doi: 10.3390/biology4040697 PMC4690014 26512705

[B179] Safarnejad M. R. Fischer R. Commandeur U. (2009). Recombinant-antibody-mediated resistance against tomato yellow leaf curl virus in nicotiana benthamiana. Arch. Virol. 154, 457–467. doi: 10.1007/s00705-009-0330-z 19234665

[B180] Saile S. C. Jacob P. Castel B. Jubic L. M. Salas-Gonzáles I. Bäcker M. . (2020). Two unequally redundant" helper" immune receptor families mediate arabidopsis thaliana intracellular" sensor" immune receptor functions. PloS Biol. 18, e3000783. doi: 10.1371/journal.pbio.3000783 32925907 PMC7514072

[B181] Salati R. (2001). Epidemiology of tomato yellow leaf curl virus in the Dominican republic and genetic analysis of genes involved in virus movement (Davis: University of California).

[B182] Salus M. S. Martin C. T. Maxwell D. P. (2007). PCR protocol for the co-dominant SCAR marker, FLUW-25, fordetection ofthe introgression at25 cM {Ty-3 locus) of chromosome 6 [En línea] En. Available at: http://www.plantpath.wisc.edu/GeminivirusResistantTomatoes/Markers/MAS-Protocols/FLUW-25FR.pdf

[B183] Sastry K. S. Zitter T. A. (2014). “Management of virus and viroid diseases of crops in the tropics,” in Plant virus and viroid diseases in the tropics (Springer), 149–480.

[B184] Sattar A. Sher A. Abourehab M. A. S. Ijaz M. Nawaz M. Ul-Allah S. . (2022). Application of silicon and biochar alleviates the adversities of arsenic stress in maize by triggering the morpho-physiological and antioxidant defense mechanisms. Front. Environ. Sci. 10. doi: 10.3389/fenvs.2022.979049

[B185] Schuster D. J. Mueller T. F. Kring J. B. Price J. F. (1990). Relationship of the sweetpotato whitefly to a new tomato fruit disorder in Florida. HortScience 25 (12), 1618–1620.

[B186] Scott J. W. Hutton S. F. Freeman J. H. (2015). Fla. 8638B and fla. 8624 tomato breeding lines with begomovirus resistance genes ty-5 plus Ty-6 and Ty-6, respectively. HortScience 50, 1405–1407. doi: 10.21273/HORTSCI.50.9.1405

[B187] Scott J. Schuster D. (1991). Screening of accessions for resistance to the Florida tomato geminivirus. Tomato Genet. Coop Rep. 41, 48–50.

[B188] Selth L. A. Randles J. W. Rezaian M. A. (2004). Host responses to transient expression of individual genes encoded by tomato leaf curl virus. Mol. Plant Microbe Interact. 17, 27–33. doi: 10.1094/MPMI.2004.17.1.27 14714865

[B189] Shankar R. Harsha S. Bhandary R. (2014). A practical guide to identification and control of tomato diseases. (India: P 24 TROPICA SEEDS PVT LTD|No 54, South End Road, 1st Floor, Nama Aurore Building, Basavangudi, Bangalore 560004). Available at: https://www.tropicaseeds.com/

[B190] Shelake R. M. Pramanik D. Kim J.-Y. (2019). Exploration of plant-microbe interactions for sustainable agriculture in CRISPR era. Microorganisms 7, 269. doi: 10.3390/microorganisms7080269 31426522 PMC6723455

[B191] Shen Q. Liu Z. Song F. Xie Q. Hanley-Bowdoin L. Zhou X. (2011). Tomato SlSnRK1 protein interacts with and phosphorylates βC1, a pathogenesis protein encoded by a geminivirus β-satellite. Plant Physiol. 157, 1394–1406. doi: 10.1104/pp.111.184648 21885668 PMC3252149

[B192] Shen X. Yan Z. Wang X. Wang Y. Arens M. Du Y. . (2020). The NLR protein encoded by the resistance gene Ty-2 is triggered by the replication-associated protein Rep/C1 of tomato yellow leaf curl virus. Front. Plant Sci. 11, 545306. doi: 10.3389/fpls.2020.545306 33013967 PMC7511541

[B193] Shuey D. J. Mccallus D. E. Giordano T. (2002). RNAi: gene-silencing in therapeutic intervention. Drug Discovery Today 7, 1040–1046. doi: 10.1016/S1359-6446(02)02474-1 12546893

[B194] Sorri V. Watanabe K. Valkonen J. (1999). Predicted kinase-3a motif of a resistance gene analogue as a unique marker for virus resistance. Theor. Appl. Genet. 99, 164–170. doi: 10.1007/s001220051221

[B195] Stenger D. C. Revington G. N. Stevenson M. C. Bisaro D. M. (1991). Replicational release of geminivirus genomes from tandemly repeated copies: evidence for rolling-circle replication of a plant viral DNA. Proc. Natl. Acad. Sci. 88, 8029–8033. doi: 10.1073/pnas.88.18.8029 1896448 PMC52439

[B196] Sunitha S. Shanmugapriya G. Balamani V. Veluthambi K. (2013). Mungbean yellow mosaic virus (MYMV) AC4 suppresses post-transcriptional gene silencing and an AC4 hairpin RNA gene reduces MYMV DNA accumulation in transgenic tobacco. Virus Genes 46, 496–504. doi: 10.1007/s11262-013-0889-z 23417222

[B197] Tabein S. Laviano L. Pecchioni N. Accotto G. P. Noris E. (2017). Pyramiding Ty-1/Ty-3 and Ty-2 in tomato hybrids dramatically inhibits symptom expression and accumulation of tomato yellow leaf curl disease inducing viruses. Arch. Phytopathol. Plant Prot. 50, 213–227. doi: 10.1080/03235408.2017.1287234

[B198] Tashkandi M. Ali Z. Aljedaani F. Shami A. Mahfouz M. M. (2018). Engineering resistance against tomato yellow leaf curl virus *via* the CRISPR/Cas9 system in tomato. Plant Signaling Behav. 13, e1525996. doi: 10.1080/15592324.2018.1525996 PMC6204811 30289378

[B199] Teixeira R. M. Ferreira M. A. Raimundo G. A. S. Fontes E. P. B. (2021). Geminiviral triggers and suppressors of plant antiviral immunity. Microorganisms 9, 775. doi: 10.3390/microorganisms9040775 33917649 PMC8067988

[B200] Thomma B. P. Nürnberger T. Joosten M. H. (2011). Of PAMPs and effectors: the blurred PTI-ETI dichotomy. Plant Cell 23, 4–15. doi: 10.1105/tpc.110.082602 21278123 PMC3051239

[B201] Valkonen J. Wiegmann K. Hämäläinen J. Marczewski W. Watanabe K. (2008). Evidence for utility of the same PCR-based markers for selection of extreme resistance to potato virus y controlled by rysto of solanum stoloniferum derived from different sources. Ann. Appl. Biol. 152, 121–130. doi: 10.1111/j.1744-7348.2007.00194.x

[B202] Vallad G. E. Messelink G. Smith H. A. (2018). “Crop protection: Pest and disease management,” in Tomatoes, 2nd ed. Ed. Euvelink E. (Boston, MA, USA: CAB International), 207–257.

[B203] Van Brunschot S. Persley D. Geering A. Campbell P. R. Thomas J. E. (2010). Tomato yellow leaf curl virus in Australia: distribution, detection and discovery of naturally occurring defective DNA molecules. Australas. Plant Pathol. 39, 412–423. doi: 10.1071/AP10083

[B204] Vanitharani R. Chellappan P. Fauquet C. M. (2005). Geminiviruses and RNA silencing. Trends Plant Sci. 10, 144–151. doi: 10.1016/j.tplants.2005.01.005 15749473

[B205] Varshney R. K. Thudi M. R. K. Börner A. (2007). Genic molecular markers in plants: development and applications. In: Varshney RK Tuberosa R (eds). Genomicsassisted crop improvement: genomics approaches and platforms (Dordrecht: Springer) 1, 13–29. doi: 10.1007/978-1-4020-6295-7_2

[B206] Verlaan M. G. Hutton S. F. Ibrahem R. M. Kormelink R. Visser R. G. Scott J. W. . (2013). The tomato yellow leaf curl virus resistance genes Ty-1 and Ty-3 are allelic and code for DFDGD-class RNA–dependent RNA polymerases. PloS Genet. 9, e1003399. doi: 10.1371/journal.pgen.1003399 23555305 PMC3610679

[B207] Verlaan M. G. Szinay D. Hutton S. F. De Jong H. Kormelink R. Visser R. G. . (2011). Chromosomal rearrangements between tomato and solanum chilense hamper mapping and breeding of the TYLCV resistance gene Ty-1. Plant J. 68, 1093–1103. doi: 10.1111/j.1365-313X.2011.04762.x 21883550

[B208] Vidavsky F. Leviatov S. Milo J. Rabinowitch H. Kedar N. Czosnek H. (1998). Response of tolerant breeding lines of tomato, lycopersicon esculentum, originating from three different sources (L. peruvianum, l. pimpinellifolium and l. chilense) to early controlled inoculation by tomato yellow leaf curl virus (TYLCV). Plant Breed. 117, 165–169. doi: 10.1111/j.1439-0523.1998.tb01472.x

[B209] Voorburg C. M. Yan Z. Bergua-Vidal M. Wolters A. A. Bai Y. Kormelink R. (2020). Ty-1, a universal resistance gene against geminiviruses that is compromised by co-replication of a betasatellite. Mol. Plant Pathol. 21, 160–172. doi: 10.1111/mpp.12885 31756021 PMC6988424

[B210] Wang Y. Jiang J. Zhao L. Zhou R. Yu W. Zhao T. (2018b). Application of whole genome resequencing in mapping of a tomato yellow leaf curl virus resistance gene. Sci. Rep. 8, 1–11. doi: 10.1038/s41598-018-27925-w 29941914 PMC6018388

[B211] Wang B. Yang X. Wang Y. Xie Y. Zhou X. (2018a). Tomato yellow leaf curl virus V2 interacts with host histone deacetylase 6 to suppress methylation-mediated transcriptional gene silencing in plants. J. Virol. 92, e00036–18. doi: 10.1128/jvi.00036-18 29950418 PMC6146709

[B212] Wolters A.-M. A. Caro M. Dong S. Finkers R. Gao J. Visser R. G. . (2015). Detection of an inversion in the Ty-2 region between s. lycopersicum and s. habrochaites by a combination of *de novo* genome assembly and BAC cloning. Theor. Appl. Genet. 128, 1987–1997. doi: 10.1007/s00122-015-2561-6 26152571 PMC4572051

[B213] Xie Z. Fan B. Chen C. Chen Z. (2001). An important role of an inducible RNA-dependent RNA polymerase in plant antiviral defense. Proc. Natl. Acad. Sci. U.S.A. 98, 6516–6521. doi: 10.1073/pnas.111440998 11353867 PMC33500

[B214] Xu Y. Crouch J. H. (2008). Marker-assisted selection in plant breeding: From publications to practice. Crop Sci. 48, 391–407. doi: 10.2135/cropsci2007.04.0191

[B215] Xu J. Xie J. Yan C. Zou X. Ren D. Zhang S. (2014). A chemical genetic approach demonstrates that MPK 3/MPK 6 activation and NADPH oxidase-mediated oxidative burst are two independent signaling events in plant immunity. Plant J. 77, 222–234. doi: 10.1111/tpj.12382 24245741 PMC4017028

[B216] Yamaguchi H. Ohnishi J. Saito A. Ohyama A. Nunome T. Miyatake K. . (2018). An NB-LRR gene, TYNBS1, is responsible for resistance mediated by the Ty-2 begomovirus resistance locus of tomato. Theor. Appl. Genet. 131, 1345–1362. doi: 10.1007/s00122-018-3082-x 29532116

[B217] Yang H. H. Xu X. Y. Jiang J. B. Li J. F. . (2016). Advanced progress on tomato yellow leaf curl disease resistance genes and disease resistance breeding. Mol Plant Breed 14, 2044–2049.

[B218] Yang X. Caro M. Hutton S. F. Scott J. W. Guo Y. Wang X. . (2014). Fine mapping of the tomato yellow leaf curl virus resistance gene Ty-2 on chromosome 11 of tomato. Mol. Breed. 34, 749–760. doi: 10.1007/s11032-014-0072-9 25076841 PMC4092234

[B219] Yang X. Guo W. Li F. Sunter G. Zhou X. (2019a). Geminivirus-associated betasatellites: Exploiting chinks in the antiviral arsenal of plants. Trends Plant Sci. 24, 519–529. doi: 10.1016/j.tplants.2019.03.010 31003895

[B220] Yang Y. Liu T. Shen D. Wang J. Ling X. Hu Z. . (2019b). Tomato yellow leaf curl virus intergenic siRNAs target a host long noncoding RNA to modulate disease symptoms. PloS Pathog. 15, e1007534. doi: 10.1371/journal.ppat.1007534 30668603 PMC6366713

[B221] Yang Y. Sherwood T. A. Patte C. P. Hiebert E. Polston J. E. (2004). Use of tomato yellow leaf curl virus (TYLCV) rep gene sequences to engineer TYLCV resistance in tomato. Phytopathology 94, 490–496. doi: 10.1094/PHYTO.2004.94.5.490 18943768

[B222] Yang X. Xie Y. Raja P. Li S. Wolf J. N. Shen Q. . (2011). Suppression of methylation-mediated transcriptional gene silencing by βC1-SAHH protein interaction during geminivirus-betasatellite infection. PloS Pathog. 7, e1002329. doi: 10.1371/journal.ppat.1002329 22028660 PMC3197609

[B223] Yang M. Zhao T. Yu W. Zhao L. (2012). New SSR marker linked to Ty-2 resistance to tomato yellow leaf curl virus. Jiangsu J. Agric. Sci. 28, 1109–1113.

[B224] Yan Z. Pérez-De-Castro A. Díez M. J. Hutton S. F. Visser R. G. Wolters A.-M. A. . (2018). Resistance to tomato yellow leaf curl virus in tomato germplasm. Front. Plant Sci. 9, 1198. doi: 10.3389/fpls.2018.01198 30177938 PMC6110163

[B225] Yan Z. Wolters A.-M. A. Navas-Castillo J. Bai Y. (2021). The global dimension of tomato yellow leaf curl disease: Current status and breeding perspectives. Microorganisms 9, 740. doi: 10.3390/microorganisms9040740 33916319 PMC8066563

[B226] Zaidi S.S.-E.-A. Tashkandi M. Mansoor S. Mahfouz M. M. (2016). Engineering plant immunity: using CRISPR/Cas9 to generate virus resistance. Front. Plant Sci. 7, 1673. doi: 10.3389/fpls.2016.01673 27877187 PMC5099147

[B227] Zakay Y. Navot N. Zeidan M. Kedar N. Rabinowitch H. Czosnek H. . (1991). Screening lycopersicon accessions for resistance to tomato yellow leaf curl virus: Presence of viral DNA and symptom development. Plant Dis. 75, 279–281. doi: 10.1094/PD-75-0279

[B228] Zamir D. Ekstein-Michelson I. Zakay Y. Navot N. Zeidan M. Sarfatti M. . (1994). Mapping and introgression of a tomato yellow leaf curl virus tolerance gene, Ty-1. Theor. Appl. Genet. 88, 141–146. doi: 10.1007/BF00225889 24185918

[B229] Zarreen F. Chakraborty S. (2020). Epigenetic regulation of geminivirus pathogenesis: A case of relentless recalibration of defence responses in plants. J. Exp. Bot. 71, 6890–6906. doi: 10.1093/jxb/eraa406 32869846

[B230] Zhang H. Gong H. Zhou X. (2009). Molecular characterization and pathogenicity of tomato yellow leaf curl virus in China. Virus Genes 39, 249–255. doi: 10.1007/s11262-009-0384-8 19590945

[B231] Zhao W. Zhou Y. Zhou X. Wang X. Ji Y. (2021). Host GRXC6 restricts tomato yellow leaf curl virus infection by inhibiting the nuclear export of the V2 protein. PloS Pathog. 17, e1009844. doi: 10.1371/journal.ppat.1009844 34398921 PMC8389846

